# Cell-Permeable Succinate Increases Mitochondrial Membrane Potential and Glycolysis in Leigh Syndrome Patient Fibroblasts

**DOI:** 10.3390/cells10092255

**Published:** 2021-08-31

**Authors:** Ajibola B. Bakare, Raj R. Rao, Shilpa Iyer

**Affiliations:** 1 Department of Biological Sciences, J. William Fulbright College of Arts and Sciences, University of Arkansas, Fayetteville, AR 72701, USA; abbakare@uark.edu; 2 Department of Biomedical Engineering, College of Engineering, University of Arkansas, Fayetteville, AR 72701, USA; rajrao@uark.edu

**Keywords:** leigh syndrome, succinate prodrug, glycolysis, TCA cycle, mitochondrial respiration

## Abstract

Mitochondrial disorders represent a large group of severe genetic disorders mainly impacting organ systems with high energy requirements. Leigh syndrome (LS) is a classic example of a mitochondrial disorder resulting from pathogenic mutations that disrupt oxidative phosphorylation capacities. Currently, evidence-based therapy directed towards treating LS is sparse. Recently, the cell-permeant substrates responsible for regulating the electron transport chain have gained attention as therapeutic agents for mitochondrial diseases. We explored the therapeutic effects of introducing tricarboxylic acid cycle (TCA) intermediate substrate, succinate, as a cell-permeable prodrug NV118, to alleviate some of the mitochondrial dysfunction in LS. The results suggest that a 24-hour treatment with prodrug NV118 elicited an upregulation of glycolysis and mitochondrial membrane potential while inhibiting intracellular reactive oxygen species in LS cells. The results from this study suggest an important role for TCA intermediates for treating mitochondrial dysfunction in LS. We show, here, that NV118 could serve as a therapeutic agent for LS resulting from mutations in mtDNA in complex I and complex V dysfunctions.

## 1. Introduction

Mitochondrial disorders are a large group of severe genetic disorders that primarily impact cells and tissues with high energy requirements [1,2]. These disorders are clinically complex, often fatal, and occur at an estimated ratio of 1 in 10,000–15,000 live births [3,4]. Leigh syndrome (LS) is a classic mitochondrial disorder that is the result of deleterious mtDNA mutations that disrupt the oxidative phosphorylation (OXPHOS) capacities of cells that are impacted [4]. Presently, there is very little evidence of targeted therapies for LS [4,5]. Recent studies has highlighted the potential for cell-permeant substrates regulating the electron transport chain (ETC) as therapeutics for mitochondrial diseases [6,7,8,9,10,11]. These substrates work by increasing tricarboxylic acid cycle (TCA) intermediates and providing alternative substrate sources for energy production in the mitochondria. One of the mitochondrial substrates that is currently being explored as a therapeutic option for LS is succinate [7,8,9,10,11,12,13]. Conversion of succinyl-CoA by the enzyme succinyl-CoA synthetase yields free succinate as an intermediate substrate of the TCA cycle, to form GTP which further donates its terminal phosphate group to ADP to form ATP [14]. Succinate is further dehydrogenated to fumarate by the flavoprotein succinate dehydrogenase (SDH), which is tightly embedded in the inner mitochondrial membrane. SDH, also known as complex II, contains a covalently bound Flavin adenine nucleotide (FAD) which gets simultaneously reduced to FADH_2_. In turn, FADH_2_ contributes to the ETC by reducing ubiquinone (CoQ) to ubiquinol (CoQH_2_). CoQ functions as an electron carrier and transfers electrons to complex III [15]. Succinate is presumed to represent one of the major sources of electrons for mitochondrial reactive oxygen species (ROS), when it is oxidized by the ETC to reduce oxygen to superoxide [16]. Succinate also contributes to the elimination of superoxide and H_2_O_2_ production [17,18], suggesting that complex II could be an important contributor for regulating ROS homeostasis. Thus, SDH couples two major pathways in the mitochondria, the TCA cycle and the ETC, both being essential for oxidative phosphorylation [19]. 

In healthy mitochondria, CI-linked respiration is largely responsible for ETC activity and subsequent ATP production by complexes I, III, IV, and V [15]. Consequently, inhibition of complex I (CI) activity can interfere with the majority of ATP production in the mitochondria. Chronic CI dysfunction leads to the disruption of NADH oxidation and cells try to compensate for the reduced NAD levels, by increasing pyruvate which gets converted to lactate, and thereby maintaining the NAD pool in the cells. Succinate offers an alternate metabolic pathway, bypassing CI-linked respiration in the case of CI defect.

Succinate is a dicarboxylic acid that takes the form of an anion in living organisms. As such, it is not cell membrane permeable and has limited uptake into cells when given exogenously [9]. To overcome this limitation, prodrugs of succinate have been developed and screened for cell permeability [9]. NV101-118 (NV118, diacetoxymethyl succinate), hereafter referred to as NV118, is one of the successful cell membrane-permeable prodrugs of succinate that was recently developed [9]. Since its development, NV118 has been explored as a potential therapeutic for various diseases [7,8,9,10,11,12,13,20]. NV118 is currently being tested for its therapeutic potential in various cellular models of diseases associated with CI dysfunction [7,8,9,10,11,20]. In all of these models, NV118 improved mitochondrial function, as evidenced by increased CII-linked respiration, membrane potential, ATP production, and a decrease in lactic acidosis [9,11,20]. Furthermore, recent studies have shown improvement in mitochondrial respiration with NV118 in cellular models with inhibition of downstream ETC enzymes such as CIV [12,13]. These studies suggest that NV118 can improve mitochondrial respiration by other means aside from elevating CII activity. 

In earlier studies in our laboratory, we reported fragmented/hyper fused mitochondrial morphology [21], abnormal ETC enzymatic activity, depressed mitochondrial function, decreased ROS, and membrane potential in fibroblast cells modeling LS. Two LS fibroblast cell lines harbored pathogenic point mutation in the mtDNA at *T8993G* in the *MTATP6* gene causing complex V deficiency, while the third LS fibroblast cell lines harbored pathogenic point mutation in the mtDNA at *T10158C* in the *MTND3* gene and the fourth LS fibroblast cell lines harbored pathogenic point mutation in the mtDNA at *T12706C* in the *MTND5* gene causing complex I deficiency. A commercially available fibroblast cell line (BJ-FB) was used as a healthy control line. 

In the present study, we used the same fibroblast cells modeling LS and the control BJ cell line and treated them for 24 h with varying concentrations of NV118 to measure changes in mitochondrial function and oxidative stress. Our results suggest that 24-hour treatment with 100 µM of NV118 can rescue some of the mitochondrial dysfunction in treated LS cells as compared with untreated cells. We report, for the time, that after a 24-hour treatment with 100 µM of NV118, cellular bioenergetics improved as a result of upregulation of glycolysis, TCA cycle, and mitochondrial membrane potential (MMP). We show that the most sustained change was in glycolysis, as mitochondrial respiration remained the same after 24-hour treatment with NV118. Together, our results suggest that NV118 could serve as a therapeutic agent for the treatment of LS.

## 2. Materials and Methods

### 2.1. Ethics Statement

This study protocol conformed to the guidelines of the Declaration of Helsinki. The current study was conducted with patient fibroblasts provided by the Medical University of Salzburg (SBG), Austria. Fibroblasts were obtained for diagnostic purposes from patients with defined disorders. Informed consent was obtained to use these samples for research in an anonymized way. In accordance with federal regulations regarding the protection of human research subjects (32 CFR 219.101(b)(4)), the University of Arkansas Office of Research Compliance determined that the project was exempt from Institutional Review Board (IRB) oversight and human research subject protection regulations.

### 2.2. NV118 Drug Preparation

The NV118 succinate prodrug (Oroboros Instruments Corporation, Innsbruck, Austria) was prepared following the manufacturer’s instructions. Aliquots of the stock were stored in a −20 °C freezer until needed for experiments. Before each experiment, a fresh working solution was prepared by adding the appropriate volume of stock solution to phenol red-free MEM. The working solution (1 mM) was prepared at 10X the final treatment concentration (100 µM). The working solutions were used up on the same day as they were prepared.

### 2.3. Cell Culture

Cultures of healthy control and four patient-derived diseased fibroblast cell lines were maintained in a fibroblast expansion medium that consisted of minimal essential medium (MEM) (Thermo Fisher Scientific, Waltham, MA, USA) supplemented with 10% fetal bovine serum (FBS) (GE Healthcare HyClone^TM^, Chicago, IL, USA) and 2 mM L-glutamine (Thermo Fisher Scientific). All cell lines were cultured and maintained at 37 °C in a humidified atmosphere of 5% CO_2_. The culture medium was replenished every two days and passaged when cells reached 80% confluence. Fibroblasts were enzymatically passaged in 0.05% Trypsin-EDTA (Thermo Fisher Scientific). All experiments were performed with cells at Passage 8 for consistency and to minimize experimental variability.

### 2.4. Mitochondrial Oxygen Consumption Detection, Glycolysis Function Test, and Bioenergetics Health Index

Cells were routinely maintained in culture following established protocols until the desired passage (Passage 8) was reached. A day before assay, 20,000 cells per well were plated and cultured in complete medium (MEM supplemented with 10% FBS and 2 mM L-glutamine). Prior to treatment, the drug treatment group had 100 μM of NV118 in basal MEM (without FBS) added to each well, while the control groups had an equivalent volume of basal MEM (without FBS) added to each well. The cells were incubated with NV118 in MEM (or basal MEM) in a 37 °C incubator with a humidified atmosphere of 5% CO_2_ for 24 h. At the end of the 24-hour incubation period, mitochondrial and glycolytic metabolic profiles were assessed following the steps below.

Changes in oxygen consumption were measured in real time using an XFe96 extracellular flux analyzer. A Seahorse XFe96 Cell Mito Stress Test Kit and glycolytic rate assay kit (Seahorse Biosciences, North Billerica, MA, USA) were used, as per the manufacturers’ instructions. Prior to use in XFe96, fibroblasts were detached using mild trypsin and seeded into the plates with a previously optimized number of 20,000 cells per well. All fibroblasts were seeded in 8–12 replicate wells per plate, with the experiment repeated at least 3–5 times.

The cells were supplemented with 180 μL Mito stress complete Seahorse medium, after which the cells were incubated in a non-CO_2_ incubator at 37 °C for one hour. Respiration was measured using the classic mitochondrial inhibitors, specific for complex I and III subunits, such as rotenone and antimycin A (0.5 μM final concentrations each). Maximum respiration was measured by the addition of an uncoupler carbonyl cyanide-4-(trifluoromethoxy) phenylhydrazone (FCCP) (0.7 μM final concentration) and oligomycin (1 μM final concentration) was added to measure proton leak. The readouts were normalized to cell numbers and analyzed using Seahorse XF96 Wave software.

We also analyzed glycolytic function in the fibroblast cell lines. A classical glycolytic rate assay was performed using the XFe96 based on the following procedure: (1) cells were cultured in buffered (5 mM HEPES buffer) Seahorse medium supplemented with glucose and pyruvate, (2) the proton efflux rate (PER) was measured after the addition of saturating amounts of glucose, (3) rotenone and antimycin A were added to inhibit mitochondrial-derived ADP phosphorylation, and (4) 2-DG was added to inhibit glycolysis. The different assay parameters, i.e., basal glycolysis, compensatory glycolysis, total proton efflux, and post 2-DG acidification, were normalized to cell number and analyzed using Seahorse XFe96 Wave software.

The mitochondrial-derived bioenergetic health index (mitoBHI), a composite index of mitochondrial quality was determined using the predefined formula: $Log\;\left(\left(mitoATP*SRC\right)/\left(proton\;leak*\;non-mito.\;resp.\right)\right)$. The glycolytic BHI (glycoBHI), an index of glycolytic respiration was determined using the formula: $Log\;\left(\left(glycoPER*Comp.\;glycolysis\right)/\left(MitoPER*\;post.2DG\;acid\right)\right)$.

### 2.5. Mitochondrial Membrane Potential Measurements

Cells were maintained in culture following established protocols until the desired passage (Passage 8) was reached. When cells reached 80–85% confluence, they were treated with either 100 μM of NV118 or an equivalent volume of basal MEM (FBS free) and incubated for 24 h. After the 24-hour incubation, mitochondrial membrane potential was evaluated following the procedure below.

On the day of the experiment, cells were enzymatically detached using 0.05% Trypsin-EDTA (Thermo Fisher Scientific) and centrifuged at 400× *g* for 5 min. Then, the cells were resuspended in basal medium, after which the desired amount of tetramethylrhodamine, ethyl ester (TMRE, Abcam, Cambridge, MA, USA) was added (for a final concentration of 50 nM). For the FCCP and oligomycin treatment groups, 20 µM and 5 µM of FCCP and oligomycin were added, respectively, for 10 min prior to treatment with TMRE. Cells were incubated with TMRE in a 37 °C 5% CO_2_ incubator for 25 min. At the end of the incubation period, cells were centrifuged at 400× *g* for 5 min. To wash off the excess dye, cells were resuspended in 1× dPBS solution and centrifuged for another 5 min. At the end of the wash, the cells were resuspended in phenol red-free basal medium and transferred to Accuri C6 plus flow cytometer (BD Biosciences, Franklin Lakes, NJ, USA) for data acquisition. 20,000 events were recorded for each cell line. After data acquisition, the data were exported as FCS files and analyzed using FlowJo_v10.6.2 software. To gate for the TMRE-positive population, cells that were not stained with TMRE were used to gate for the TMRE negative cell populations. The mean fluorescent intensity (MFI) values, a measure of the geometric mean of TMRE positive cells was obtained for statistical analysis.

### 2.6. Intracellular ROS Measurement

Cells were maintained in culture following established protocols until the desired passage (Passage 8) was reached. A day before assay, 20,000 cells per well were plated and cultured in complete medium (MEM supplemented with 10% FBS and 2 mM L-glutamine). In addition, the treatment groups had either 100 μM NV118 or an equivalent volume of basal MEM (FBS free) in each well. After 24 h, the intracellular ROS levels were measured using the 2′,7′-dichlorofluorescein diacetate (DCFDA/H2DCFDA) a cell-permeable cellular ROS assay kit (Abcam, Cambridge, MA, USA). This dye measures hydroxyl (-OH), peroxyl (O22-), and other reactive oxygen species (ROS). Within the cells, DCFDA is hydrolyzed by nonspecific esterases to release DCF, which is readily oxidized by intracellular ROS. The oxidized product emits green fluorescence at (Ex/Em = 485/535). Following the manufacturer’s instructions, the cells were incubated with 10 μM of DCFDA. The cells were incubated in the dark for 45 min with this dye. As a positive control, 200 μM of TBHP was added for 60 min before DCFDA treatment. At the end of the incubation period with DCFDA, the cells were washed once with phenol red-free MEM to get rid of the excess dye. Finally, phenol red-free MEM was added to each well, and cells were transferred to a plate reader (BioTek, Winooski, VT, USA) for data acquisition. The fluorescent intensity was background corrected and adjusted by subtracting fluorescent intensity from blank wells.

### 2.7. Statistical Analysis

In order to ensure scientific rigor and reproducibility, for the bioenergetics analysis, an ANOVA design accounting for 4–5 biological and 10–12 technical replicates from the control (BJ-FB) and diseased (SBG1-FB, SBG2-FB, SBG4-FB, and SBG5-FB) cells that were nested within treated and untreated groups was used to identify any differences with respect to control BJ-FBs. Post hoc Tukey HSD tests were used to identify differences among specific groups. Data are presented as the mean ± standard deviation (SD) and were analyzed using the GraphPad Prism 8 software (GraphPad Software, San Diego, CA, USA). A *p* < 0.05 was considered to be significant. 

Similarly, for the ROS analysis, an ANOVA design accounting for 3 biological and 10–12 technical replicates from control (BJ-FB) and diseased (SBG1-FB, SBG2-FB, SBG4-FB, and SBG5-FB) cells that were nested within treated and untreated groups was used to identify any differences with respect to control BJ-FBs. Post hoc Tukey HSD tests were used to identify differences among specific groups. Data are presented as the mean ± standard deviation (SD) and were analyzed using the GraphPad Prism 8 software (GraphPad Software, San Diego, CA, USA). A *p* < 0.05 was considered to be significant.

For the MMP analysis, an ANOVA design accounting for 3 biological and 3–4 technical replicates from control (BJ-FB) and diseased (SBG1-FB, SBG2-FB, and SBG4-FB, SBG5-FB) cells that were nested within treated and untreated groups was used to identify any differences with respect to the control BJ-FB. Post hoc Tukey HSD tests were used to identify differences among specific groups. Data are presented as the mean ± standard deviation (SD) and were analyzed using the GraphPad Prism 8 software (GraphPad Software, San Diego, CA, USA). A *p* < 0.05 was considered to be significant.

## 3. Results

### 3.1. Effect of NV118 on Cell Viability and Mitochondrial Respiration in Non-Permeabilized (Intact) Control BJ-FB

**Figure 1:**
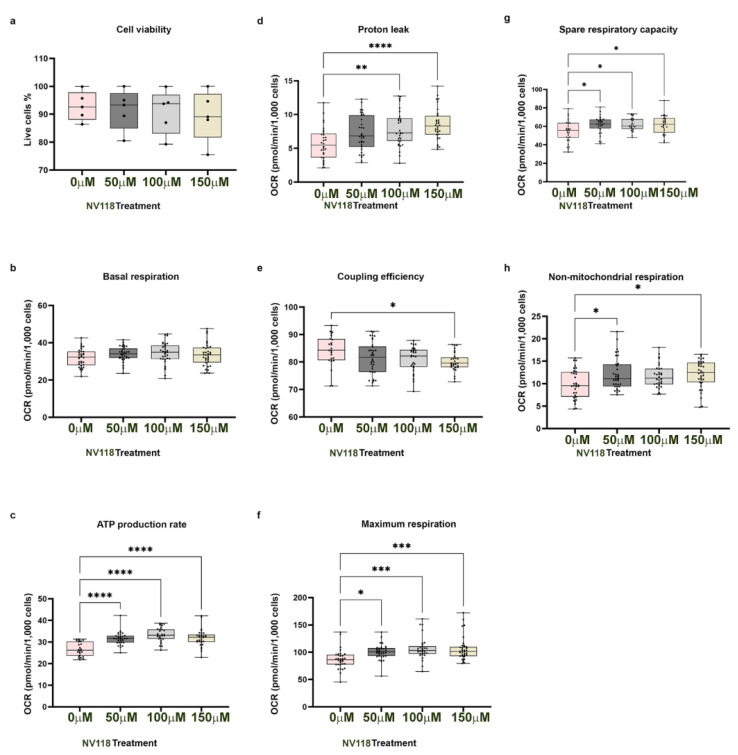
Cell viability and mitochondria respiration profile of CTL BJ-FB after NV118 treatments. The control BJ-FBs were treated with 0, 50, 100, or 150 µM of NV118 for 30 min, and (**a**) cell viability was assessed. Oxygen consumption rate (OCR) under basal conditions and after the addition of NV118 was measured (**b**–**f**). The following respiration parameters were recorded: (**b**) basal respiration, (**c**) ATP production rate (oligomycin-sensitive), (**d**) proton leak, (**e**) coupling efficiency, (**f**) maximal respiration, (**g**) spare respiratory capacity, and (**h**) nonmitochondrial respiration after Rot/AA injection. All parameters are in pmol/min/1000 cells. Data are mean +/− SD. Experiments were repeated at least three times on different days under the same conditions. * *p* < 0.05, ** *p* < 0.01, *** *p* < 0.001, and **** *p* < 0.00001. Pink, dark gray, light gray, and tan bars represents treatment with 0 µM (vehicle), 50, 100, and 150 µM of NV118, respectively.

First, we assessed the effect of different concentrations of NV118 on the viability of intact control BJ-FB cells. Cell viability was measured following a 30 min exposure to 50, 100, or 150 µM of NV118. As a control, phenol red-free MEM (vehicle) was added to the wells without NV118 drug treatment. To ensure scientific rigor and reproducibility, the analysis was conducted on the control cell lines (n = 5) at passage eight. The results indicate no significant difference between the NV118 treated and untreated control BJ-FB (Figure 1a).

Next, we assessed mitochondrial respiration in intact control BJ-FB using the same NV118 concentrations and vehicle. Oxygen consumption rate (OCR) using a Seahorse XFe96 flux analyzer was measured after acute treatment with 50, 100, and 150 µM of NV118 or vehicle. Basal respiration was recorded before administration of the different concentrations of NV118. Subsequently, other oxidative phosphorylation properties were measured including proton leak, maximal respiration, and nonmitochondrial respiration, after sequential injections of ATP synthase inhibitor oligomycin, the uncoupler carbonyl cyanide-4-(trifluoromethoxy) phenylhydrazone FCCP, complex I inhibitor rotenone, and complex III inhibitor antimycin A into the wells.

As expected, before NV118 was administered into the cell culture plates, the basal respiration for the control BJ-FB was similar between all treatment groups (Figure 1b). The addition of NV118 resulted in a dose-dependent response of the oxidative phosphorylation properties recorded. The results show a significant increase (*p* < 0.0001) in ATP production (Figure 1c) in the NV118 treatment groups. The highest increase in ATP production was observed in the BJ-FB with 100 µM NV118 treatment (23%), while both 50 and 150 µM NV118 treatment resulted in a 19% increase in ATP production relative to the untreated group. Although NV118 resulted in a significant increase in ATP rate, we also observed a significant increase (*p* < 0.01) in proton leak (Figure 1d) with 100 µM (by 39%) and 150 µM (by 49%) NV118 treatment relative to the untreated group. While the leak was not significantly different in the 50 µM treatment group, proton leak increased by 26% relative to the untreated group. The increase in leak suggests that NV118 treatment results in mild uncoupling of the mitochondrial membrane. Indeed, there was a 3% and 4% decrease in coupling efficiency (Figure 1e) for the 50 µM and 100 µM NV118 treatments, respectively. While the decrease in coupling efficiency was not significant in this treatment group, 150 µM NV118 treatment significantly decreased (*p* < 0.05) coupling efficiency by 5% as compared with the untreated group. The maximum respiration rate (Figure 1f) caused by the addition of FCCP showed a significant increase of 16% (*p* < 0.05) in 50 µM, 23% (*p* < 0.001) increase in 100 µM, and 22% (*p* < 0.001) increase in 150 µM NV118 treatment groups relative to the untreated BJ-FB. The observed significant increase in maximal respiration upon treatment suggests that NV118 increases the cells’ ability to rapidly oxidize substrates when the metabolic need arises. 

In ongoing work, we have shown that spare respiratory capacity (SRC) is an important bioenergetics variable required by cells to adapt and respond to ATP demand (Supplementary Figure S1). The result in this study showed a significant increase (*p* < 0.05) in SRC values (Figure 1g) by 13% in the 50 µM, and by 14% in the 100 and 150 µM treatment groups as compared with the untreated group. The observed significant increase in SRC indicates that NV118 treatment could help BJ-FB cells adapt when there is a sudden increase in ATP demand. Finally, the mitochondrial respiration was inhibited by simultaneously treating cells with rotenone and antimycin A. Nonmitochondrial respiration, which is typically attributed to the non-ETC oxidases present in the cell [22] was significantly higher in the 50 µM (20%) and 150 µM (22%) treatment groups relative to the untreated cell lines (Figure 1h). However, nonmitochondrial respiration was not significantly different when BJ-FBs were treated with 100 µM of NV118. 

On the basis of the dose response and effects on nonmitochondrial respiration (Figure 1h), ATP production (Figure 1c), and the SRC (Figure 1g), 100 µM NV118 was selected as the optimal concentration for further evaluation on the diseased cell lines.

### 3.2. The Glycolytic Pathway Is Upregulated in the Control BJ-FBs after 24-Hour Treatment with NV118

One of the hallmarks of LS is lactic acidosis resulting from the conversion of pyruvate to lactate to maintain the NAD+ pool [9]. The result from the optimization studies led to the hypothesis that NV118 improves mitochondrial function and decreases the dependence of the cells on lactate production. Since succinate is a substrate that connects the TCA cycle with the ETC, we also wanted to understand how NV118 affects the TCA cycle and cellular respiration. Therefore, cellular glycolysis was examined to understand the impact of NV118 on this metabolic pathway. Using a Seahorse XFe96 flux analyzer, proton efflux rate (PER) rate was measured in the control BJ-FB after a 24-hour treatment with 100 µM of NV118 or an equivalent amount of vehicle (phenol red-free MEM). At the end of this assay, basal glycolysis, glycolysis-derived ATP, glycolytic capacity, and non-glycolytic respiration values were obtained. To ensure scientific rigor and reproducibility, analysis was conducted on the control cell line (n = 3) at passage eight.

**Figure 2:**
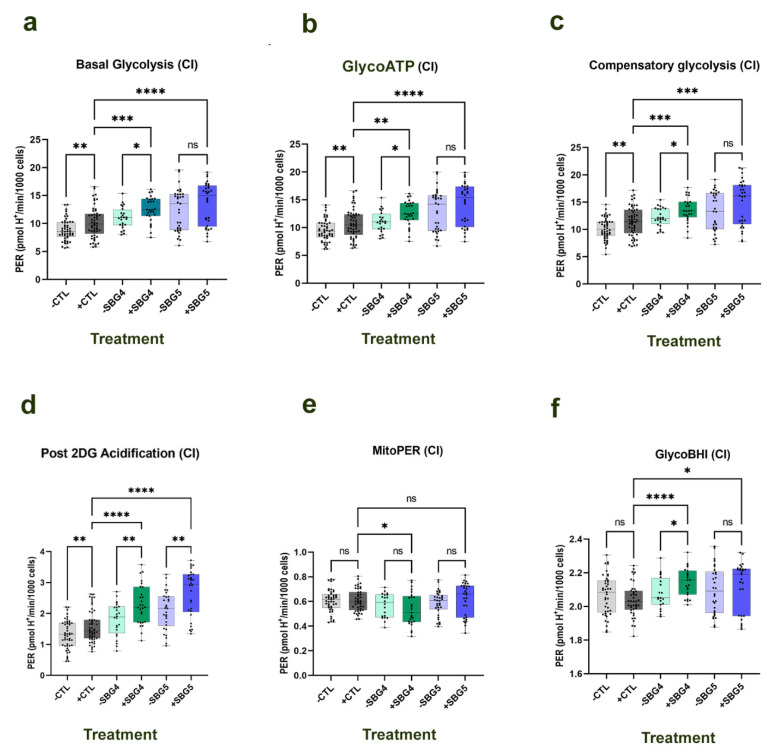
Glycolytic respiration profile of SBG4-FB (*T10158C*) and SBG5-FB (*T12706C*) cell lines after NV118 treatment. The control BJ-FB, SBG4-FB (*T10158C*), and SBG5-FB (*T12706C*) were treated with 100 µM of NV118 or vehicle (phenol red-free MEM) for 24 h, and the proton efflux rate (PER) was measured at the end of the 24-hour treatment period. Cell line showing (**a**) basal glycolysis; (**b**) GlycoATP production rate; (**c**) compensatory glycolysis after blocking ETC using Rot/AA; (**d**) post 2DG acidification (non-glycolytic acidification); (**e**) mitoPER; (**f**) glycoBHI. All parameters are in pmol H^+^/min/1000 cells. Data are mean +/− SD. Experiments were repeated at least three times on different days under the same conditions. * *p* < 0.05, ** *p* < 0.01, *** *p* < 0.001, and **** *p* < 0.0001. ns = Not significant. The light gray bars represent treatment of BJ-FB, light green bars represent treatment of SBG4-FB (*T10158C*), light purple bars represent treatment of SBG5-FB (*T12706C*) (with vehicle (-NV118); dark gray bars represent treatment of BJ-FB, dark green bars represent treatment of SBG4-FB (*T10158C*), dark purple bars represent treatment of SBG5-FB (*T12706C*), with 100 µM of NV118 (+NV118).

The results show a significant increase (*p* < 0.01) by 15% in basal glycolysis (Figure 2a) in the control BJ-FB upon NV118 treatment as compared with the untreated group. This increase corresponded to a significant (13%, *p* < 0.01) increase in the ATP derived (Figure 2b) from glycolysis in the BJ-FB cell upon NV118 treatment. When the mitochondria were inhibited with rotenone and antimycin A to drive the cells’ dependence on glycolysis, glycolytic capacity (Figure 2c) significantly increased (*p* < 0.01) by 13% in the NV118 treatment group relative to the untreated group. The addition of the glycolysis inhibitor, 2-deoxyglucose resulted in a significantly higher PER, as measured by an increase in post-2DG acidification (Figure 2d) in the BJ-FB (*p* < 0.01, 20%) NV118 treatment group as compared with the untreated group. Taken together, these results indicate that NV118 treatment increased utilization of TCA substrate; hence, the glycolytic pathway had to be upregulated to make pyruvate and acetyl-CoA shuttle into the TCA cycle. The slight increase in mitoPER (by 2%) recorded in the BJ-FB (Figure 2e) with NV118 treatment further supports this hypothesis. To better understand the relationship between disease and bioenergetic markers of mitochondrial dysfunction, a value concept termed “bioenergetics health index or BHI” has been proposed for measuring overall mitochondrial dysfunction [23,24]. The mitoBHI value based on mitochondrial bioenergetics parameters captures positive aspects of bioenergetics function (SRC and ATP-linked respiration) and contrasts with potentially deleterious aspects (nonmitochondrial oxygen consumption and proton leak). Since the fibroblasts also demonstrated reliance on glycolysis, we designated “glycoBHI” to capture positive aspects of glycolysis (basal PER, compensatory glycolysis) and contrasted these with potentially deleterious non-glycolytic parameters (mitoPER and post 2DG acidification). We observed a 2% decrease in glycoBHI (Figure 2f), further strengthening the possibility that NV118 upregulates the glycolytic pathway to contribute towards production of TCA substrates.

### 3.3. Treatment with NV118 for 24 h Significantly Increased Glycolysis in the LS Cells Harboring mtDNA Mutation T10158C in the MTND3 Gene but Not in the LS Cells with mtDNA Mutation T12706C in the MTND5 Gene Affecting CI Function

Having examined the effect of NV118 on the control BJ-FB, we proceeded to analyze the effect on diseased cell lines. Next, we examined the two cell lines modeling LS harboring pathogenic mutant DNA in *MT-ND3* (SBG4-FB (*T10158C*)) and *MT-ND5* (SBG5-FB (*T12706C*)) gene causing a complex I defect. A glycolytic rate assay was performed after 24-hour treatment with 100 µM or NV118 or equivalent amount of vehicle (phenol red-free MEM). The same glycolytic parameters were recorded as those of the BJ-FB. 

Basal glycolysis (Figure 2a) was higher by 13% (*p* < 0.05) and 9% in SBG4-FB (*T10158C*) and SBG5-FB (*T12706C*), respectively, when NV118 was added. The glycolytic ATP production rate (glycoATP) was also higher in SBG4-FB (*T10158C*) (by 15%, *p* < 0.01) and SBG5-FB (*T12706C*) (9%) (Figure 2b) cell lines in the NV118 treatment relative to the untreated group. When the mitochondria were inhibited with rotenone and antimycin A to induce glycolysis, the glycolytic capacity also increased in SBG4-FB (*T10158C*) (11%, *p* < 0.05) in the presence of NV118 (Figure 2c). Although not statistically significant, glycolytic capacity increased in SBG5-FB (*T12706C*) (12%) (Figure 2c) in the presence of NV118. The addition of the glycolysis inhibitor, 2-deoxyglucose resulted in a significant increase (*p* < 0.01) in post 2DG acidification in SBG4-FB (*T10158C*) (27%), and SBG5-FB (*T12706C*) (27%) (Figure 2d) in the NV118 treatment as compared with the untreated samples. The increase in post 2DG acidification after addition of NV118 indicates the possibility of other pathways not attributed to glycolysis and is worthy of future investigation. Similar to the observation in the control BJ-FB, NV118 treatment increased utilization of TCA substrates; hence, leading to the upregulation of the glycolytic pathway in these cell lines. Although not statistically significant, the 5% increase in mitoPER recorded in the SBG5-FB (*T12706C*) (Figure 2e) with NV118 treatment supports the increased activity of TCA cycle enzymes. In SBG4-FB (*T10158C*) cells with NV118 treatment, the results showed a decreased trend in mito PER values (Figure 2e). We observed a trend towards a higher glycoBHI (Figure 2f) in the SBG4-FB (*T10158C*) cells (3% increase) when NV118 was present, while the glycoBHI stayed relatively the same in both the SBG5-FB (*T12706C*) treated and untreated samples (Figure 2f). Although NV118 treatment upregulated glycolysis in both cell lines, NV118 treatment in SBG4-FB (*T10158C*) does not result in a corresponding increase in TCA cycle substrate production, evidenced by the lower mitoPER (Figure 2e). Conversely, the upregulated glycolysis in SBG4-FB (*T10158C*) with NV118 treatment could be contributing to lactic acid production or other unknown pathways. 

Interestingly, we also observed differences between control BJ-FB and SBG4-FB (*T10158C*) and SBG5-FB (*T12706C*) upon NV118 treatment. These comparisons are indeed interesting as it demonstrates that the diseased SBG4-FB (*T10158C*) and SBG5-FB (*T12706C*) can activate multiple pathways. Our results show that basal glycolysis (Figure 2a), glycoATP (Figure 2b), compensatory glycolysis (Figure 2c), post 2DG acidification (Figure 2d), and glycoBHI (Figure 2f) are all significantly increased in both the NV118-treated SBG4-FB (*T10158C*) and SBG5-FB (*T12706C*) as compared with the control BJ-FB lines. Basal glycolysis (Figure 2a) was higher by 23% (*p* < 0.001) and 33% (*p* < 0.0001) in SBG4-FB (*T10158C*) and SBG5-FB (*T12706C*), respectively, as compared with BJ-FB upon NV118 treatment. Glycolytic ATP production rate (glycoATP) was also higher in SBG4-FB (*T10158C*) (by 23%, *p* < 0.01) and SBG5-FB (*T12706C*) (32%, *p* < 0.0001) (Figure 2b) cell lines as compared with BJ-FB upon NV118 treatment. When the mitochondria were inhibited with rotenone and antimycin A to induce glycolysis, glycolytic capacity also increased in SBG4-FB (*T10158C*) (18%, *p* < 0.001) and SBG5-FB (*T12706C*) (30%, *p* < 0.001) (Figure 2c) as compared with BJ-FB upon NV118 treatment. The addition of the glycolysis inhibitor, 2-deoxyglucose resulted in a significant increase (*p* < 0.0001) in post 2DG acidification in SBG4-FB (*T10158C*) (45%), and SBG5-FB (*T12706C*) (69%) (Figure 2d) as compared with BJ-FB upon NV118 treatment. The increase in post 2DG acidification after addition of NV118 indicates the possibility of other pathways not attributed to glycolysis and is worthy of future investigation. We also observed a significant increase in glycoBHI in both SBG4-FB (*T10158C*) (by 4%, *p* < 0.0001) and SBG5-FB (*T12706C*) (1%, *p* < 0.05) (Figure 2f) as compared with BJ-FB upon NV118 treatment. Taken together, the results from the glycolytic respiration suggest that the location of the subunit affected by each mtDNA mutation influences the metabolic response with NV118 treatment.

### 3.4. NV118 Did Not Significantly Increase Glycolysis in LS Cells Harboring Pathogenic mtDNA Mutation (T8993G) in the MTATP6 Gene Affecting Complex V

Most studies on administering succinate prodrug NV118 have focused on using it as therapy for disorders resulting from CI deficiencies containing nuclear DNA mutations [8,9,10,11]. However, some recent studies have suggested improvement in mitochondrial function when NV118 was used to treat disorders resulting from CIV dysfunction [12,13]. Since glycolysis was upregulated in the control BJ-FB without ETC defects, we wanted to investigate the effect of NV118 on glycolysis in LS cell lines with mutations affecting CV (ATP synthase) of the ETC. For this purpose, two LS patient fibroblast cell lines carrying point mutation *T8993G* in the *MT-ATP6* gene (SBG1-FB (*T8993G*) with >96% heteroplasmy and SBG2-FB (*T8993G*) with >91% heteroplasmy) were selected for these experiments. Following the same approach as before, glycolysis rate assays were estimated after a 24-hour treatment with 100 µM of NV118 or equivalent amount of vehicle (phenol red-free MEM) in all the cell lines.

**Figure 3:**
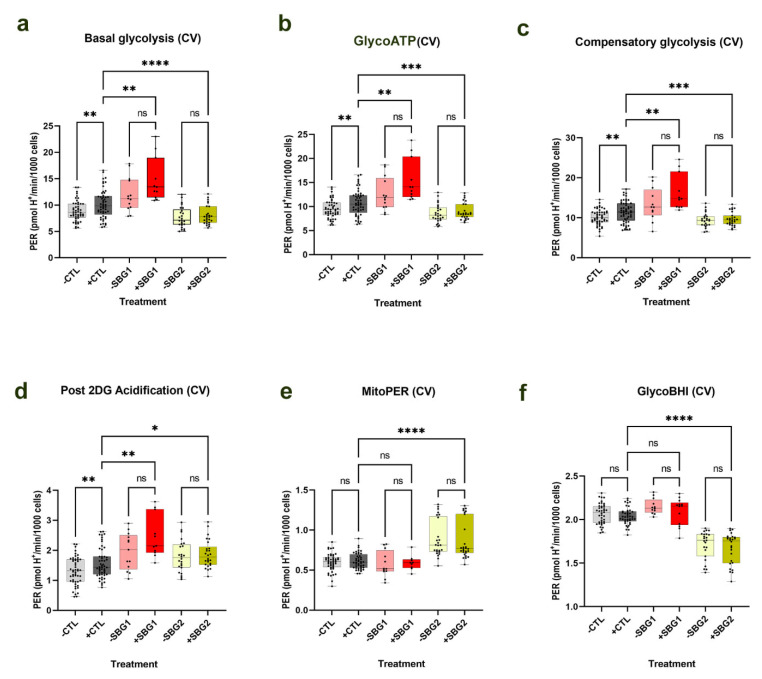
Glycolytic respiration profile of SBG1-FB (*T8993G*) and SBG2-FB (*T8993G*) cell lines after NV118 treatment. SBG1-FB (*T8993G*) and SBG2-FB (*T8993G*) were treated with 100 µM or NV118 or vehicle (phenol red-free MEM) for 24 h, and the proton efflux rate (PER) was measured at the end of the 24-hour treatment period. Cell line showing (**a**) basal glycolysis; (**b**) GlycoATP production rate; (**c**) compensatory glycolysis after blocking ETC using Rot/AA; (**d**) post 2DG acidification (non-glycolytic acidification); (**e**) mitoPER; (**f**) glycoBHI. All parameters are in pmol H^+^/min/1000 cells. Data are mean +/− SD. Experiments were repeated at least three times on different days under the same conditions. * *p* < 0.05, ** *p* < 0.01, *** *p* < 0.001, and **** *p* < 0.0001. The light gray bars represent treatment of BJ-FB, pink bars represent treatment of SBG1-FB (*T8993G*), light green bars represent treatment of SBG2-FB (*T8993G*) (with vehicle (-NV118); dark gray bars represent treatment of BJ-FB, red bars represent treatment of SBG1-FB (*T8993G*), and dark green bars represent treatment of SBG2-FB (*T8993G*) with 100 µM NV118 (+NV118).

There was statistically no significant difference in any of the parameters (Figure 3) recorded for both SBG1-FB-*T8993G* and SBG2-FB-*T8993G* in the presence of NV118 as compared with the vehicle MEM treated cells (denoting −NV118), although SBG1-FB *T8993G* and SBG2-FB-*T8993G* cells showed trends towards an upregulation in the glycolytic pathway. In the SBG1-FB-*T8993G* cells, basal glycolysis, glycoATP, glycolytic capacity, and post 2DG acidification (Figure 3a–d) showed increasing trends by 24%, 23%, 23%, and 25%, respectively, in the NV118 treatment group relative to the untreated group. However, the increase was modest in the SBG2-FB-*T8993G* cell lines. We recorded 5%, 4%, 2%, and 4% increases in basal glycolysis, glycoATP, glycolytic capacity, and post 2DG acidification (Figure 3a–d), respectively, in the SBG2-FB-*T8993G* cell line after NV118 treatment. The mitoPER (Figure 3e) in SBG1-FB-*T8993G* exhibited a (2% increase) similar to what was estimated for SBG5-FB-*T12706C* cells, while SBG2-FB-*T8993G* (Figure 3e) exhibited a (2% decrease) similar to what was observed for SBG4-FB-*T10158C* cells. Overall, the glycoBHI for SBG1-FB-*T8993G* decreased by (3%) (Figure 3f) and decreased by (2%) in SBG2-FB-*T8993G* cells (Figure 3f) treated with NV118 as compared with the untreated group.

### 3.5. 24-Hour Treatment with NV118 Does Not Induce Cellular ROS Production in LS or Control Fibroblast Cells

Next, we investigated the effects of NV118 on cellular ROS production in control and fibroblast cells modeling LS, and harboring pathogenic mtDNA variants (*T8993G*, *T10158C*, and *T12706C*), in *MTATP6*, *MTND3,* and *MTND5* genes leading to complex V and I defect and depressed ATP production. Previous reports have shown that supplying mitochondria with a substantial increase in succinate concentration results in reverse electron transport (RET), consequently, increasing reactive oxygen species (ROS) production [25,26,27]. Therefore, we wanted to test whether the four vulnerable LS cells treated with 100 µM of NV118 (succinate) for 24 h would generate reactive oxygen species in the presence of NV118. LS and BJ control FB cells were grown to 80% confluency and treated with 20 µM of DCFDA/H_2_DCFDA (2′,7′-dichlorofluorescein diacetate) and vehicle for 24 h (see Materials and Methods for details). The following day, a BioTek plate reader was used to record the fluorescent intensity for each cell line. Data were background corrected to reduce background noise from excitation and emission crosstalk. DCFDA is a fluorogenic probe that diffuses freely into cells. When inside a cell, cellular esterases, in the cells, deacetylate this probe, converting it into a non-fluorescent compound. In the presence of hydroxyl, peroxyl, and other reactive oxygen species (ROS), DCFDCA gets oxidized to a highly fluorescent compound, DCF (2′,7′-dichlorofluorescein) which can be detected by a plate reader with excitation/emission at 485 nm/535 nm. 

**Figure 4:**
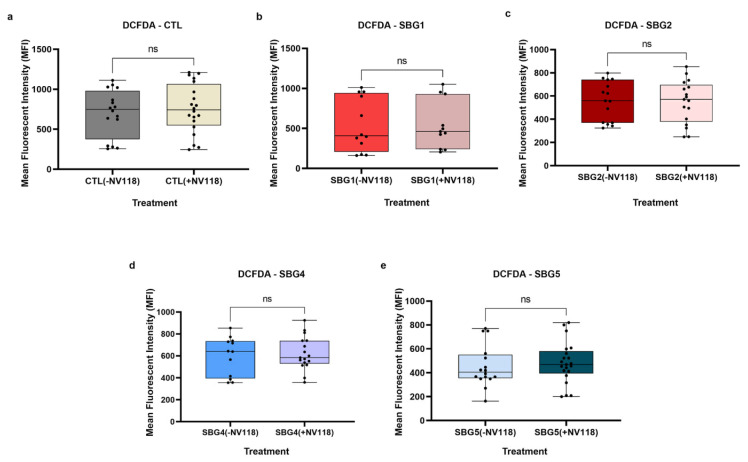
Cellular ROS production in CTL BJ-FB and LS diseased fibroblast. All cell lines were treated with 100 µM of NV118 or vehicle (phenol red-free MEM) for 24 h, and cellular ROS was measured at the end of the 24-hour treatment period using DCFDA/H_2_DCFDA. Cellular ROS in (**a**) control CTL BJ-FB and cell lines with (**b**,**c**) CV defect (SBG1-FB (*T8993G*) and SBG2-FB (*T8993G*)), and (**d**,**e**) CI defect with and without NV118 treatment. Data are mean +/− SD. Experiments were repeated at least three times on different days under the same conditions. ns- not significant.

The results indicate that there was no significant difference in ROS production between the NV118 treated and untreated groups in all cell lines (Figure 4). It has been reported previously that high concentrations of succinate could exert inhibitory effects on mitochondrial respiration [19,28]. Furthermore, high concentrations of succinate could result in upregulation of RET, contributing to an increase in ROS production and subsequent cellular apoptosis [19,26,27]. Treatment with NV118 did not result in any significant changes to cellular ROS production in either the control BJ-FB or any of the diseased cell lines. As indicated above, we expected that if NV118 results in RET, we would observe a significant increase in ROS production in all cell lines after NV118 treatment. Since we did not observe an increase in ROS in the presence of NV118, this indicates that the NV118 concentration (100 µM) used in this study did not oversaturate the quinone pool (Q-pool) or increase RET in either the LS or control cell lines. Given that mutations resulting in disturbances to CII activity have been reported to result in ROS production as well [17], our result further suggests that the addition of NV118 did not negatively affect the functions of CII.

### 3.6. Mitochondrial Membrane Potential Significantly Improved in SBG4-FB (T10158C) and SBG5-FB (T12706C) Exhibiting CI Defect and SBG2-FB (T8993G) with CV Defect after 24-Hour Treatment with NV118

Respiratory complex II oxidizes succinate to fumarate as part of the citric acid cycle and reduces ubiquinone to ubiquinol in the ETC. Previous experimental evidence suggested that prodrug NV118 (complex II substrate, succinate) did not play a significant role in the production of physiological or pathological intracellular reactive oxygen species in LS or control BJ cells. Therefore, we sought to understand the effects of prodrug NV118 treatment on mitochondrial membrane potential (MMP) in fibroblast cells and BJ control cells harboring pathogenic mtDNA (four LS: SBG1-FB (*MT-ATP6*-*T8993G*), SBG2-FB (*MTATP6*-*T8993G*), SBG4-FB (*MTND3-T10158C*), and SBG5-FB (*MT-ND5-T12706C*) one CTL, BJ-FB). All cells were treated with 100 μM of NV118 or vehicle (phenol red=free MEM) for 24 h and flow cytometry methods were used to measure MMP with TMRE (tetramethylrhodamine, ethyl ester) (see Materials and Methods for details). TMRE is a positively charged dye that is attracted to the negative potential across the inner mitochondrial membrane, and thus accumulates in functionally active mitochondria in live cells [29]. In active mitochondria, TMRE is sequestered in the matrix because of the negative charge in the matrix of these mitochondria. Depolarized or inactive mitochondria are not able to sequester TMRE as the MMP is compromised in these mitochondria. In this study, MMP was measured as mean fluorescent intensity (MFI) in all cell lines. 

**Figure 5:**
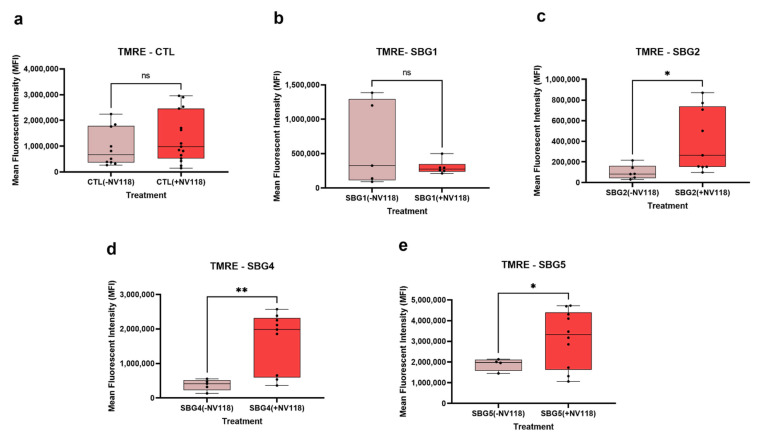
Mitochondrial membrane potential (MMP) analysis CTL BJ-FB and LS diseased fibroblast. Using flow cytometry, along with membrane-potential sensitive dye (TMRE), the MMP was evaluated for (**a**) CTL-BJ-FB, (**b**) SBG1-FB (**c**) SBG2-FB, (**d**) SBG4-FB and (**e**) SBG5-FB. Mean fluorescence intensity (MFI) was calculated based on three independent runs and is shown for each cell sample. The panels show comparisons between NV118 treated and untreated groups when stained with TMRE only. * *p* < 0.05 and ** *p* < 0.01. The light red bars represent treatment with vehicle (−NV118), while dark red bars represent NV118 treatment groups (+NV118).

The results from this study showed that upon treatment with prodrug NV118, SBG2-FB (*T8993G*)*,* SBG4-FB (*T10158C*), and SBG5-FB (*T12706C*) cells exhibited a significant increase in MMP by 306% in SBG2-FB (*T8993G*) (*p* < 0.05) (Figure 5c), 329% in SBG4-FB (*T10158C*) (*p* < 0.01) (Figure 5d), and 66% in SBG5-FB (*T12706C*) (*p* < 0.05) (Figure 5e) as compared with the untreated group. MMP stayed the same in the control BJ-FB (Figure 5a) and SBG1-FB (*T8993G*) (Figure 5b) cell lines between the treated and untreated groups. This result suggests that prodrug NV118 increased electron flux and proton translocation into the mitochondrial intermembrane space (IMS) in the SBG2-FB (*T8993G*), SBG4-FB (*T10158C*), and SBG5-FB (*T12706C*), cell lines. The data is consistent with other reports elsewhere that have shown that the addition of prodrug NV118 increased MMP, indicative of mitochondrial oxidation of succinate via succinate dehydrogenase (complex II) [9]. 

When the uncoupler, cyanide *p*-trifluoromethoxyphenylhydrazone (FCCP), was added to depolarize the mitochondria, all cell lines responded to this treatment by exhibiting a decreasing trend in MMP relative to the TMRE only treatment (Supplementary Materials Figures S2–S4c,d and Figures S3 and S4g,h). However, significant difference was observed only in SBG4-FB-*T10158C* (*p* < 0.01) and SBG5-FB-*T12706C* (*p* < 0.01) cell lines. While TMRE + FCCP resulted in depolarization in all of the cell lines, a comparison between the untreated and NV118 treated groups showed that the SBG2-FB (*T8993G*) cells (Supplementary Materials Figure S3e) revealed an additional defect apart from complex V deficiency. In our previous studies, we reported that SBG2-FB (*T8993G*) has an uncoupling defect [21] because the cells were unresponsive to FCCP treatment. In this study, we observed that in the NV118 untreated group, MMP was indistinguishable between the TMRE only and TMRE + FCCP treatment group (Supplementary Materials Figure S3g). Upon addition of NV118, there was an overall increase in MMP between the TMRE and TMRE + FCCP treatment groups, indicating succinate induced depolarization of the inner mitochondrial membrane upon addition of uncoupler-FCCP in SBG2-FB (*T8993G*) cells (Supplementary Materials Figure S3h). This result suggests that the mutations in the SBG2-FB (*T8993G*) cells also affect the ability of this cell line to properly oxidize substrates.

Finally, we evaluated MMP in the presence of oligomycin. As expected, the addition of oligomycin resulted in hyperpolarization in all the cell lines in the NV118 untreated group (Supplementary Materials Figures S2–S4c and Figures S3 and S4g). In the presence of prodrug NV118 and oligomycin, the mitochondria became depolarized in most of the cell lines (Supplementary Materials Figures S2–S4d and Figures S3 and S4h). This is evident by the significant decrease in MMP in the SBG2-FB (*T8993G*) (by 67%, *p* < 0.05) (Supplementary Materials Figure S3f), SBG4-FB (*T10158C*) (by 60%, *p* < 0.0001) (Supplementary Materials Figure S4b), and SBG5-FB (*T12706C*) (by 65%, *p* < 0.01) (Supplementary Materials Figure S4f), and the trend towards a decrease in control BJ-FB (by 31%) (Supplementary Materials Figure S2b) between the NV118 treated and untreated groups. This observation could be attributed to proton leaking caused by inhibition of the ATP synthase. To maintain substrate oxidation by complex II when the ATP synthase activity is inhibited, some of the protons could be leaking into the matrix to maintain the electron flux. Interestingly, there were no change in MMP in SBG1-FB (*T8993G*) cells (Supplementary Materials Figure S3b) after the addition of oligomycin.

### 3.7. Mitochondrial Bioenergetics Was Not Altered in LS Cell Lines after 24-Hour Treatment with Prodrug NV118

Next, we hypothesized that an increase in MMP would contribute to mitochondrial respiration in the diseased LS cell lines. To test this hypothesis, a mitochondrial stress test was performed on the four LS cell lines and the control BJ-FB. Using a Seahorse XFe96 flux analyzer, the oxygen consumption rate was measured in all cell lines after a 24-hour treatment with 100 µM or NV118 or without NV118 (added equivalent amount of phenol red-free MEM) as vehicle controls. The following oxidative phosphorylation parameters were recorded: basal respiration, leak, maximal respiration, and nonmitochondrial respiration, after sequential injections of ATP synthase inhibitor oligomycin, the uncoupler carbonyl cyanide-4-(trifluoromethoxy) phenylhydrazone FCCP, complex I inhibitor rotenone, and complex III inhibitor antimycin A into the wells. The analysis was conducted in all cell lines (n = 3–4) at passage eight. We have previously observed that spare respiratory capacity (SRC) is an important variable in determining how flexible a cell is in responding to changes in energetic demand (Supplementary Materials Figure S1). Therefore, in addition to reporting the mitochondrial-derived ATP rate, we also examined the rate of SRC in all cell lines with or without NV118 treatment. The results suggest that NV118 did not significantly improve mitochondrial respiration in any of the cell lines (Supplementary Materials Figures S5–S9). This result is not consistent with other reports that have shown that NV118 treatment improves mitochondrial respiration [9,10] Although we observed improvement in mitochondrial respiration (Figure 1b–h) in the optimization experiments during acute 20-minute exposure of prodrug NV118 in the control BJ-FB, the result was different during the 24-hour treatment. It is conceivable that once the prodrug NV118 (succinate) is oxidized to fumarate, malate, and oxaloacetate, the cells are not able to sustain the CII-linked mitochondrial respiration, because several reports have shown oxaloacetate competitively inhibits succinate dehydrogenase [30,31,32].

## 4. Discussion

**Figure 6:**
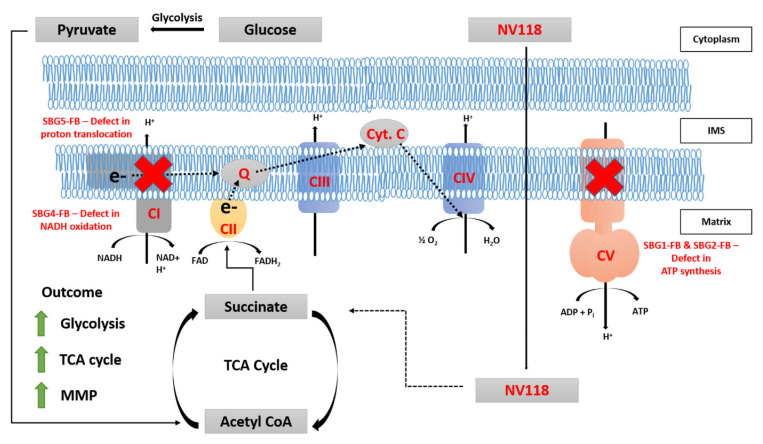
Summary figure describing the mechanism of action of NV118. NV118 is a succinate prodrug that is membrane permeable. In the mitochondria, NV118 (succinate) is oxidized by succinate dehydrogenase (CII). Under physiological conditions, both CI and CII contribute electrons (e-) to the quinone (Q) pool. Electrons from the Q pool are shuttled to CIII, and then these electrons are donated to cytochrome c (Cyt. C) before they finally get donated to CIV when the final electron acceptor, oxygen (O_2_), accepts these electrons and becomes reduced to water (H_2_O). In addition to electron transport, CI, CIII, and CIV also translocate protons (H^+^) from the matrix into the intermembrane space (IMS). This proton gradient is used by CV to generate ATP. Under physiological conditions, CI-linked respiration is favored, however, when CII substrates are in abundance, CII-linked respiration becomes predominant. Since CII cannot shuttle protons, substrate oxidation increases to maintain MMP. Furthermore, to prevent the accumulation of oxaloacetate, a potent inhibitor of CII, glycolysis is upregulated. Together, these processes drive an increase in TCA cycle enzyme activity. The red signs show that the mtDNA mutations in SBG1-FB (*T8993G*) and SBG2-FB (*T8993G*) result in CV dysfunction, while mutations in SBG4-FB (*T10158C*) and SBG5-FB (*T12706C*) result in CI dysfunction. After 24-hour treatment with 100uM of NV118, a succinate prodrug, we observed an increase in glycolysis, MMP, and TCA cycle activity.

To date, there is no cure for patients affected with LS, with fatality in patients usually within a decade after their initial diagnosis [33,34]. Recently, cell-permeant ETC substrates have been proposed as therapeutics for various ETC defects [9,10]. In these studies, succinate prodrugs have been used as a bypass for disorders involving deficiencies in complex I. The succinate prodrugs have also been used to rescue mitochondrial disorders associated with a defect in complex IV, an ETC complex whose activity is downstream of that of complex I [12,13]. It is important to note that these studies have looked at acute effects of NV118 treatment to improve respiration and have not fully examined the long-term effects of NV118 on different biochemical deficiencies. So far, none of the studies have used succinate prodrugs for alleviating symptoms of LS harboring pathogenic mtDNA mutations in the *MTATP6* gene affecting complex V and the *MTND3* and *MTND5* genes affecting complex I. Therefore, we wanted to understand the mechanisms (Figure 6) as well as alleviate the bioenergetic defects by providing succinate, as a therapeutic drug (NV118) in the vulnerable LS cell lines containing pathogenic mtDNA mutations affecting complexes I and complex V. In addition, our study focused on evaluating the medium/long term effects of NV118, which allowed us to better distinguish the direct and indirect effects upon NV118 treatment, without increasing the risk of ROS production.

It has been reported previously that high concentrations of succinate exert inhibitory effects on mitochondrial respiration [19,28]. Furthermore, high concentrations of succinate could result in upregulation of RET, contributing to an increase in ROS production and subsequent cellular apoptosis [19,26,27]. Therefore, optimization experiments for estimating drug toxicity were performed in control BJ-FB cells. The addition of different concentrations of NV118 (50, 100, and 150 μM) to the BJ-FB cells showed that the 50 μM and 100 μM NV118 treatment groups led to a 1% and 2% decrease in cell viability, respectively, while 150 mM led to a 4% decreased in cell viabilityas compared with the non-treated group. These results led us to conclude that 100 μM of NV118 would be tolerated by the disease lines as well, and therefore was used in subsequent respiration experiments. We observed the highest increase in ATP production in the group treated with 100 μM of NV118, which correlated with a significant increase in maximal respiration and SRC as well. It is worth noting that some of the increases in oxygen consumption with higher concentrations of NV118 (150 μM), contributed to a dose-dependent elevation of proton leak and lowered coupling efficiency in control BJ-FB relative to the untreated group. Therefore, 150 μM of NV118 was not selected for further studies. Together, the cell viability and the mitochondrial respiration studies convinced us that 100 μM of NV118 was the optimal concentration for evaluating the effects of the drug on mitochondrial function.

The results of the respiration experiments in BJ-FB indicated a significant increase (by 18%, *p* < 0.05) in nonmitochondrial respiration in cells treated with 100 μM of NV118 as compared with the untreated group. Nonmitochondrial respiration is typically attributed to the non-ETC oxidase present in the cell [22], suggesting reduced involvement of non-ETC oxidases. Subsequently, glycolysis was evaluated after a 24-hour treatment with 100 μM of NV118. In the TCA cycle, oxidation of succinate results in the production of fumarate. Fumarate is further oxidized to malate, and malate becomes oxidized to produce oxaloacetate. In the absence of succinate, succinate dehydrogenase (SDH CII) is inhibited and deactivated by oxaloacetate [31,35,36]. As a prodrug of succinate, the addition of NV118 for 20 min resulted in a short-term increase in CII respiration, which could result in accumulation of TCA cycle intermediates upstream of succinate. One of those intermediates is oxaloacetate (OAA), and if not used up it continues to accumulate, and could result in inhibition of CII activity. Therefore, we hypothesized that the addition of NV118 would result in the upregulation of glycolysis. In support of this hypothesis, upon NV118 treatment, we observed a significant increase (*p* < 0.05) in basal glycolysis and glycoATP production rate in the control BJ-FB and the LS SBG4-FB (*T10158C*) cell line with complex I defect, with an upward trend in glycolytic respiration in the other LS cell lines, SBG1-FB (*T8993G*), SBG2-FB (*T8993G*), and SBG5-FB (*T12706C*). When glycolysis was inhibited with 2-deoxyglucose (2DG), there was a significant increase (*p* < 0.01) in the control BJ-FB and a trend towards an increase in post 2DG acidification in all the diseased FB cell lines. Since the addition of 2DG inhibits glycolysis, it prevents lactate from being formed. However, during cellular respiration, the CO_2_ produced during oxidation reactions by TCA cycle dehydrogenases contributes to further acidification [35,36]. This further supports the hypothesis that NV118 upregulates glycolysis to enhance the production of other TCA cycle substrates. 

It is well known that reverse electron transport (RET) via complex I and NAD^+^ dependent pathways occurs when there is an overreduction of the CoQ pool by electrons from CII [25,26,27,37]. To exclude the possibility that the treatment with NV118 was causing RET and contributing to reactive oxygen species production, we examined intracellular ROS levels using DCFDA/H_2_DCFDA, a fluorescent precursor, after 24-hour treatment with 100 µM of NV118. This dye measures hydroxyl, peroxyl, and other ROS within cells. DCFDA is hydrolyzed by esterases to release DCF, which is readily oxidized by intracellular ROS, thus, emitting a green fluorescence (Ex 475/Em 515). The results from the ROS studies supported the observations made during the optimization studies with control BJ-FB cells. The treatment with 100 µM of NV118 did not result in a significant difference in intracellular ROS levels in any of the cell lines between the NV118 treated and untreated groups. The intracellular ROS levels observed in this study are consistent with our previous results, where LS diseased cell lines exhibited lower mitoROS levels as compared with the control BJ-FB cells (Supplementary Materials Figure S10). We conclude that the specific concentration of (succinate) produg NV118 used in this study did not contribute to complex I and NAD^+^ mediated reverse electron transport (RET) or increase intracellular ROS levels in either the control or LS cell lines.

Knowing that the increase in CII respiration did not result in elevated RET or ROS levels, next, we examined the MMP levels in the control and diseased FB in the presence and absence of prodrug NV118. As expected, we observed a significant increase (*p* < 0.05) in MMP levels in both complex I defective LS (SBG4-FB (*T10158C*) and SBG5-FB (*T12706C*)) cells upon NV118 treatment as compared with the untreated group. We also observed a significant increase (*p* < 0.05) in MMP levels in SBG2-FB (*T8993G*) with CV defect after NV118 addition. In the control BJ-FB cells, treatment with NV118 led to a 39% increase (although not significant) in MMP levels relative to the untreated cells. In the SBG1-FB (*T8993G*), the MMP levels stayed the same in both the NV118 treated and untreated groups. When we supplemented cells with uncouplers (FCCP) and ATPase inhibitor (oligomycin) to both treated and untreated groups to depolarize and hyperpolarize MMP, respectively, FCCP resulted in depolarization, as predicted. Interestingly, supplementation of NV118 to SBG2-FB (*T8993G*) cells, rescued the mitochondrial defect. We have reported previously that SBG2-FB (*T8993G*) has an additional uncoupling defect [21]. However, addition of exogenous substrate (NV118) allowed for complete depolarization (significant decrease in MMP, *p* < 0.001) when cells were supplemented with the uncoupler FCCP. When oligomycin was added, we predicted hyperpolarization in the NV118 untreated group. The results showed that all of the cell lines in the treated group, except SBG1-FB (*T8993G*), exhibited membrane depolarization instead of hyperpolarization. Since oligomycin is an ATPase inhibitor, it could eliminate the influence of mitochondrial ATP hydrolysis due to the decrease observed in membrane potential [38]. In intact cells, all mitochondria possess an endogenous proton leak, which may serve an important purpose in completing the proton circuit in the absence of ATP synthesis [39]. This could plausibly explain the depolarization of the membrane observed in this study.

In quantifying mitochondrial respiration to check if the increased MMP corresponded with increased OCR levels, we observed that mitochondrial respiration was unchanged after 24-hour treatment with NV118. However, in our optimization studies with NV118 for 20 min in the control BJ-FB cells, the OCR measurements showed a significant increase (*p* < 0.05) in mitoATP production, maximal respiration, and SRC rates. Therefore, this surprising result shows that the 20-minute treatment with NV118 can result in a transient increase in mitochondrial respiration, while the 24-hour treatment resulted in no change in mitochondrial respiration in all cell lines. It is possible that a 24-hour treatment with prodrugs NV118 led to increased TCA cycle substrates. Alternately, there was an initial increase in flux to produce ATP, which was transported out into the cytoplasm by unknown mechanisms to increase glycolysis and maintain the energy requirement of the cells. What we observed after the 24-hour treatment was an increase in aerobic and an increase in the MMP levels that corresponded to the effect of the 100 μM concentration of the prodrug NV118. The results from this study suggest other mechanisms of action for the succinate prodrug NV118. Ongoing studies in our laboratory are focused on metabolomic analyses to further provide information on the mechanisms of action of energy maintenance pathways and the role of TCA-cycle metabolites in these cell lines. 

Other studies using NV118 as a therapy for CI-deficient cells, have suggested that the drug works by bypassing CI and upregulating CII respiration [9,11,20]. While this explanation is valid in cell lines with CI deficiencies, other labs have also shown improvement in mitochondrial function in cells with deficiencies in other ETC enzymes downstream of CI [12,13]. In this study, bypassing CI with NV118 does not fully explain how the disease phenotype is rescued in cells exhibiting CI and CV deficiencies. The results from this study indicate upregulation of glycolysis and an increase in MMP without increasing intracellular ROS levels. 

It has been demonstrated elsewhere that activated macrophages undergo metabolic alterations to support their proinflammatory functions [40]. In these studies, stimulation of macrophages with LPS (lipopolysaccharides), endotoxins found in the outer membrane of Gram-negative bacteria, resulted in a switch to succinate-dependent respiration, and a subsequent increase in glycolysis and MMP. In macrophages, the increased succinate oxidation resulted in ROS production and production of proinflammatory cytokines. While the addition of prodrug NV118 did not increase intracellular ROS levels in LS cell lines, we observed similar increases in aerobic glycolytic flux and MMP, consistent with other studies [40]. We postulate that the addition of succinate (NV118) increases glycolysis over a 24-hour period to prevent the accumulation of oxaloacetate (a potent inhibitor of CII) [31,41]. The trend towards increased MitoPER and elevated post 2DG acidification in most of the cell lines in the presence of NV118, further supports this hypothesis. Since CO_2_ from the TCA cycle can contribute to acidification [35], when glycolysis is inhibited by 2DG, the other source of acidification could be from the TCA cycle. Upregulation of glycolysis by NV118 increases TCA cycle intermediates (Figure 6) and also contributes to ATP production [42]. It is useful to note that succinate itself is a product of a substrate-level phosphorylation step in the TCA cycle [19]. Therefore, increasing TCA cycle intermediates could help provide additional ATP in cell lines with mutations affecting OXPHOS capacities.

LS is a disorder with no known cure. According to the results from this study, we have also shown, for the first time, that in addition to rescuing mitochondrial dysfunction in cell lines with mutations impacting CI, NV118 has the potential to rescue mitochondrial dysfunction in cell lines with mutations impacting CV (ATP synthase). Although previous studies have focused on improving mitochondrial respiration, we show, here, that perhaps instead of trying to improve OXPHOS capacities in patients with LS, alternate therapies that focus on providing important TCA cycle intermediates could prove to be more beneficial. This is the first study that shows a beneficial effect in four LS lines harboring mtDNA mutations (*T8993G*, *T10158C*, and *T12706C*) without increasing the risk of ROS production in the 24-hour exposure to NV118. Since there was no observed change in ROS production between control and LS-FBs, it indicates that the optimal concentration of NV118 should be well tolerated in cells/tissues of patients with LS. Other studies have, however, shown that by exposing cells to saturating levels of succinate, a part of the electron flow is reversed leading to RET and increased generation of mitochondrial ROS [43]. In the context of possible treatment options for LS, we have shown that increasing glycolysis is important because glucose metabolism produces useful intermediates for other metabolic pathways, such as synthesis of amino acids or fatty acids. An interesting aspect of glycolysis and NV118 is that upon NV118 hydrolysis, formaldehyde is released which, at high concentration, has an inhibitory effect on glycolysis [44]. However, in our studies, we have demonstrated that the optimized 100 μM of NV118 exposure for 24 h with minimal effect on cell viability and ROS does not appear to inhibit glycolysis. Our study has also demonstrated an improvement in mitochondrial function, based on an increase in MMP upon NV118 treatment. Taken together, these results point to a potential therapeutic effect of NV118 for LS. Therefore, a better understanding of the dynamic nature of metabolic compensatory mechanisms is important to address in the context of developing novel therapeutic strategies for primary mitochondrial diseases such as LS [45]. 

The results from this study have opened an avenue for further questions to be addressed in the future. For instance, what intervals of treatment are ideal to observe the full beneficial effect of NV118 in LS patients? In this study, all the LS cell lines used were derived from patients with early-onset LS. Clinical reports and metadata analysis are starting to suggest a difference in disease presentation and prognosis for patients with early-onset and late-onset LS [46,47]. Perhaps, including cell lines derived from patients with late-onset LS presentation could provide insights into how late-onset LS disease would respond to prodrug NV118 treatment. Nevertheless, this is the first study showcasing a single therapeutic dose of NV118 in LS patient cells harboring pathogenic mtDNA mutations in the *MTATP6*, *MTND3*, and *MTND5* genes affecting complexes I and V of the electron transport chain.

## 5. Conclusions

In summary, we report, for the first time, that NV118 shows a therapeutic effect in four LS lines harboring mtDNA mutations. Our results demonstrate that, in addition to rescuing mitochondrial dysfunction in cell lines with mutations impacting CI, NV118 has the potential to rescue mitochondrial dysfunction in cell lines with mutations impacting CV. We showed that 24-hour treatment with NV118 resulted in increased glycolysis and MMP while reducing intracellular ROS levels. Although previous studies have focused on improving mitochondrial respiration, this study shows that perhaps instead of trying to improve OXPHOS capacities in patients with LS, alternate therapies that focus on providing important TCA cycle intermediates could prove to be more beneficial.

## Data Availability

No dataset has been deposited in a repository, and the data from the studied patient fibroblasts are not publicly available, in agreement with privacy law and our institutional policies.

## Supplementary Materials

The following are available online at https://www.mdpi.com/article/10.3390/cells10092255/s1, Figure S1: Correlation plot between Spare Reserve Capacity (SRC) and mitochondrial ATP (mitoATP) production, Figure S2: Mitochondrial membrane potential (MMP) analysis of BJ-FB with and without NV118, Figure S3: Mitochondrial membrane potential (MMP) analysis of SBG1-FB (T8993G) and SBG2-FB (T8993G) with and without NV118, Figure S4: Mitochondrial membrane potential (MMP) analysis of SBG4-FB (T10158C) and SBG5-FB (T12706C) with and without NV118, Figure S5: Mitochondrial respiration profile of CTL BJ-FB with and without NV118, Figure S6: Mitochondrial respiration profile of SBG1-FB (T8993G) with and without NV118, Figure S7: Mitochondrial respiration profile of SBG2-FB (T8993G) with and without NV118, Figure S8: Mitochondrial respiration profile of SBG4-FB (T10158C) with and without NV118, Figure S9: Mitochondrial respiration profile of SBG5-FB (T12706C) with and without NV118, Figure S10: Correlation plot between mitochondrial ROS and cellular ROS.
