# Supplementary material for: Cell-Permeable Succinate Increases Mitochondrial Membrane Potential and Glycolysis in Leigh Syndrome Patient Fibroblasts
Source: Cells. 2021 Aug 31;10(9):2255. doi: 10.3390/cells10092255 (PMC8470843; doi:10.3390/cells10092255)
Supplement: Supplementary file 1 [file cells-10-02255-s001.zip › cells-1322026-supplementary.pdf]

cells MDPI

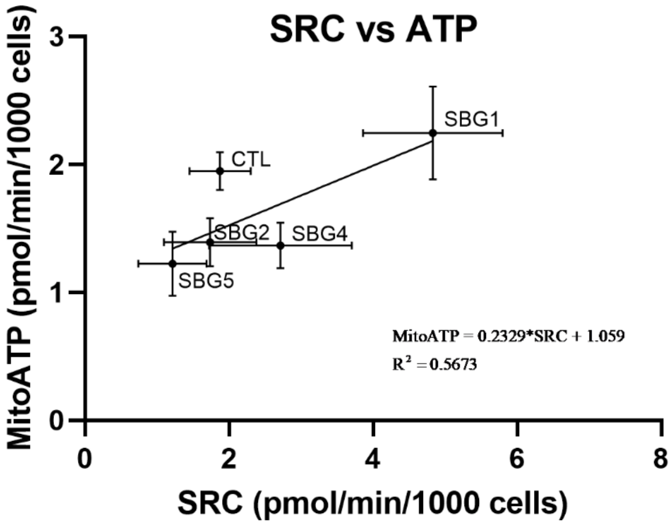


**Supplementary Figure S1.** Correlation plot between Spare Reserve Capacity (SRC) and mitochondrial ATP (mitoATP) production. As noted, spare respiratory capacity (SRC) is an important bioenergetics variable required by cells to adapt and respond to mitoATP demand. Correlation data is plotted for all five lines (CTL, SBG1-FB, SBG2-FB, SBG4-FB, SBG5-FB).

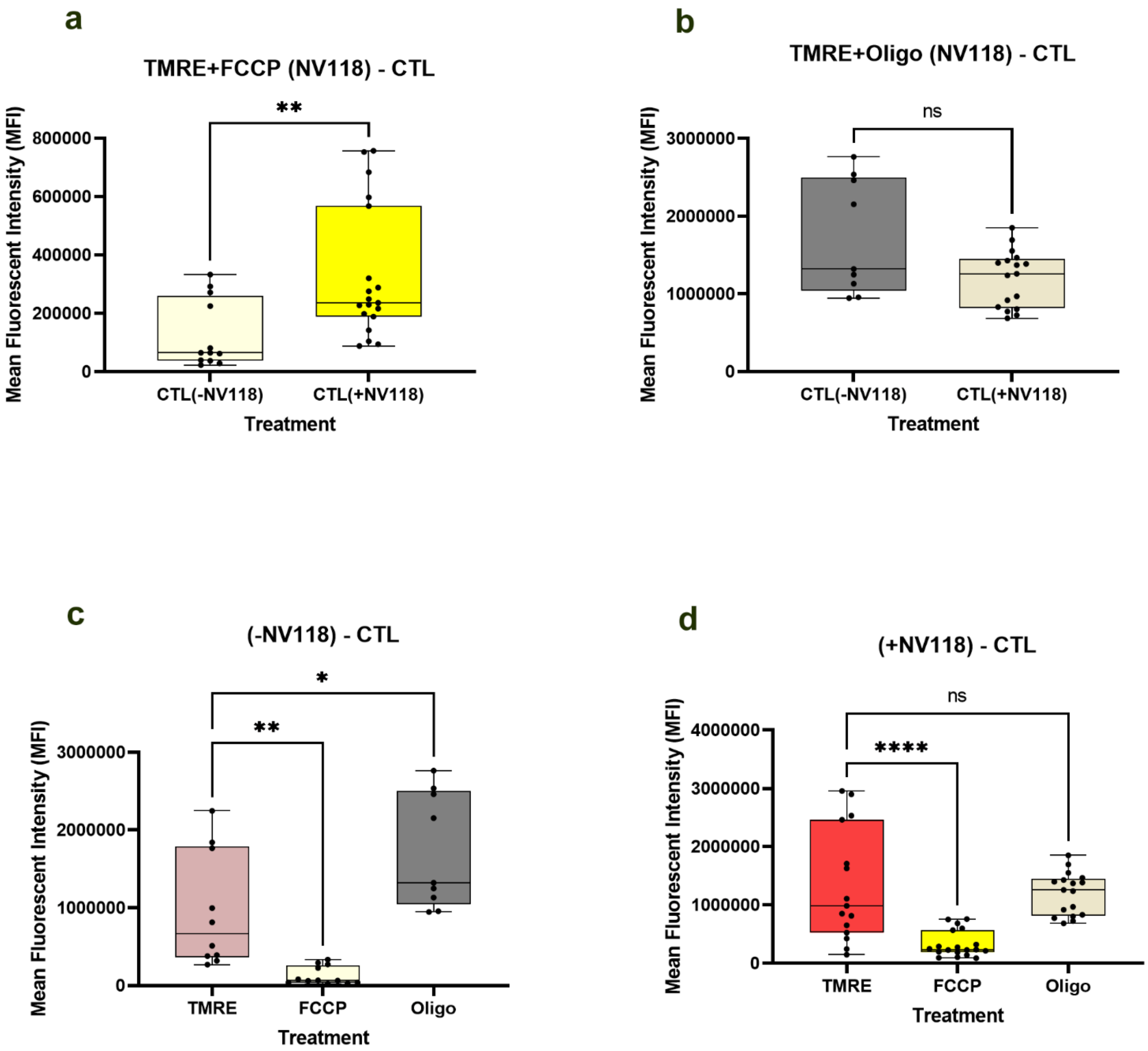


**Supplementary Figure S2. Mitochondrial membrane potential (MMP) analysis of BJ-FB with and without NV118.** Using flow cytometry, along with membrane-potential sensitive dye (TMRE), MMP was evaluated. Mean fluorescence intensity (MFI) was calculated based on three independent runs and is shown for CTL BJ-FB. Comparisons between NV118 treated and untreated groups when (a) stained with TMRE+FCCP and (b) stained with TMRE+oligomycin. Comparison between TMRE only, TMRE+FCCP, and TMRE+oligomycin in the (c) NV118 untreated groups and (d) NV118 treated group. *p<0.05** p<0.01*** p<0.001 ****p<0.00001. Light red, yellow, and dark gray bars represent treatment with vehicle (-NV118). While dark red, yellow, and tan bars represent NV118 treatment groups (+NV118).

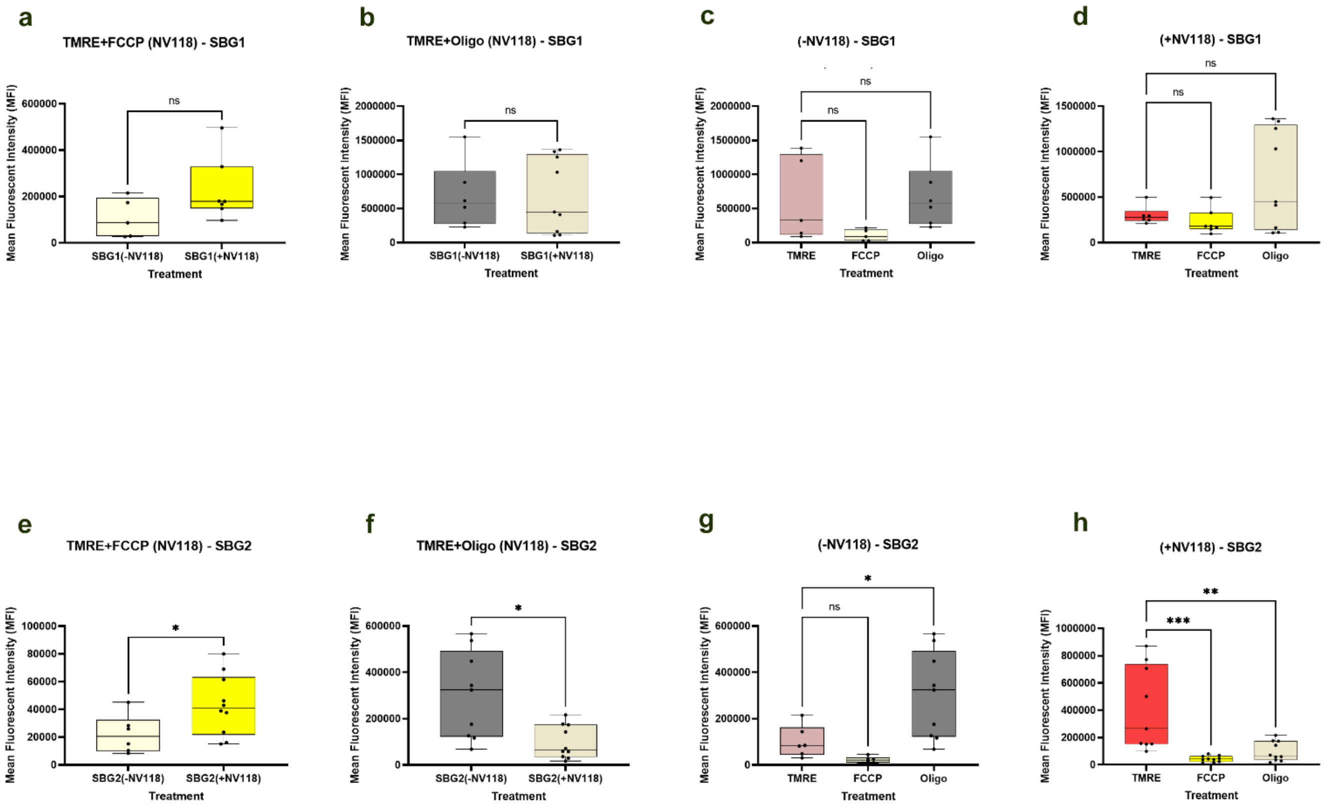


**Supplementary Figure S3. Mitochondrial membrane potential (MMP) analysis of SBG1-FB (*T8993G*) and SBG2-FB (*T8993G*) with and without NV118.** Using flow cytometry, along with membrane-potential sensitive dye (TMRE), MMP was evaluated. Mean fluorescence intensity (MFI) was calculated based on three independent runs and are shown for SBG1-FB (*T8993G*) and SBG1-FB (*T8993G*). Comparisons between NV118 treated and untreated groups when (a, e) stained with TMRE+FCCP and (b,f) stained with TMRE+oligomycin. Comparison between TMRE only, TMRE+FCCP, and TMRE+oligomycin in the (c,g) NV118 untreated groups and (d,h) NV118 treated group. *p<0.05** p<0.01*** p<0.001 ****p<0.00001. Light red, yellow, and dark gray bars represent treatment with vehicle (-NV118). While dark red, yellow, and tan bars represent NV118 treatment groups (+NV118).

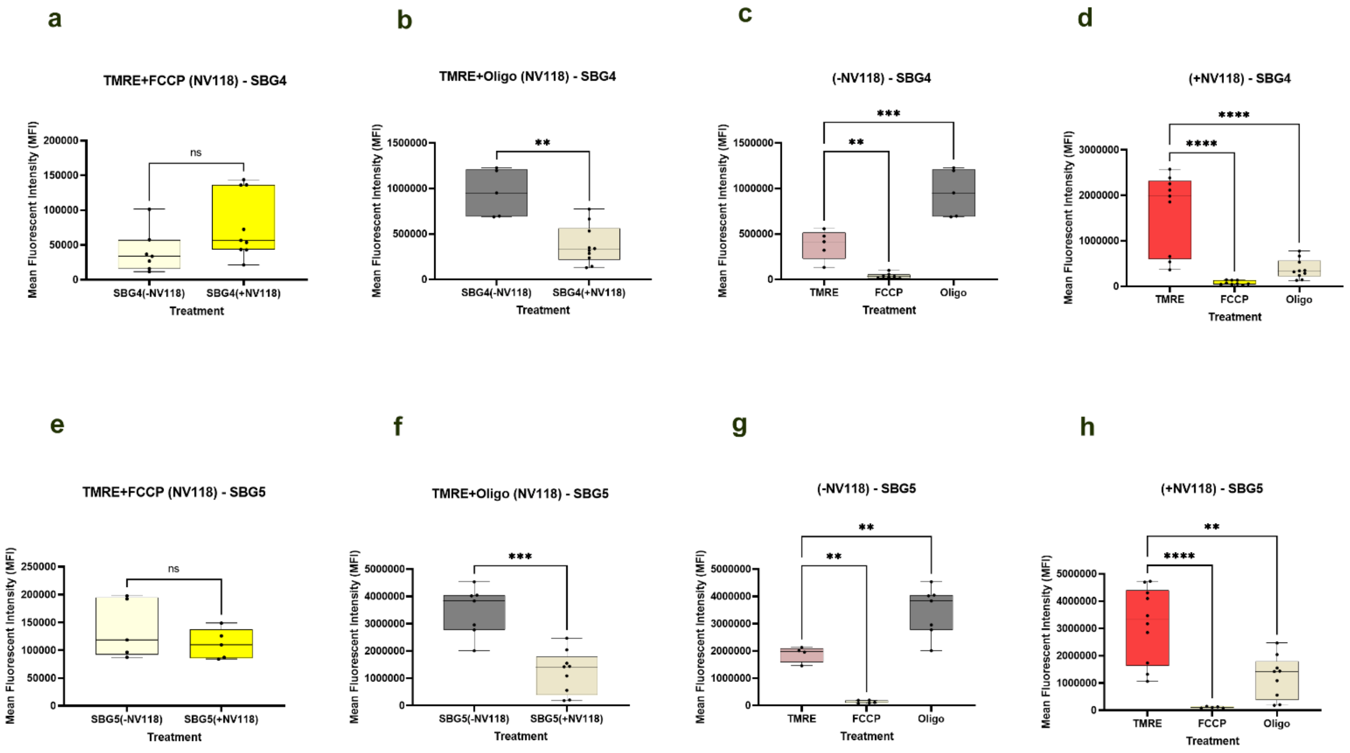


**Supplementary Figure S4. Mitochondrial membrane potential (MMP) analysis of SBG4-FB (*T10158C*) and SBG5-FB (*T12706C*) with and without NV118.** Using flow cytometry, along with membrane-potential sensitive dye (TMRE), MMP was evaluated. Mean fluorescence intensity (MFI) was calculated based on three independent runs and are shown for SBG4-FB (*T10158C*) and SBG5-FB (*T12706C*). Comparisons between NV118 treated and untreated groups when (a, e) stained with TMRE+FCCP and (b,f) stained with TMRE+oligomycin. Comparison between TMRE only, TMRE+FCCP, and TMRE+oligomycin in the (c,g) NV118 untreated groups and (d,h) NV118 treated group. *$p<0.05$** $p<0.01$*** $p<0.001$ ****$p<0.00001$. Light red, yellow, and dark gray bars represent treatment with vehicle (-NV118). While dark red, yellow, and tan bars represent NV118 treatment groups (+NV118).

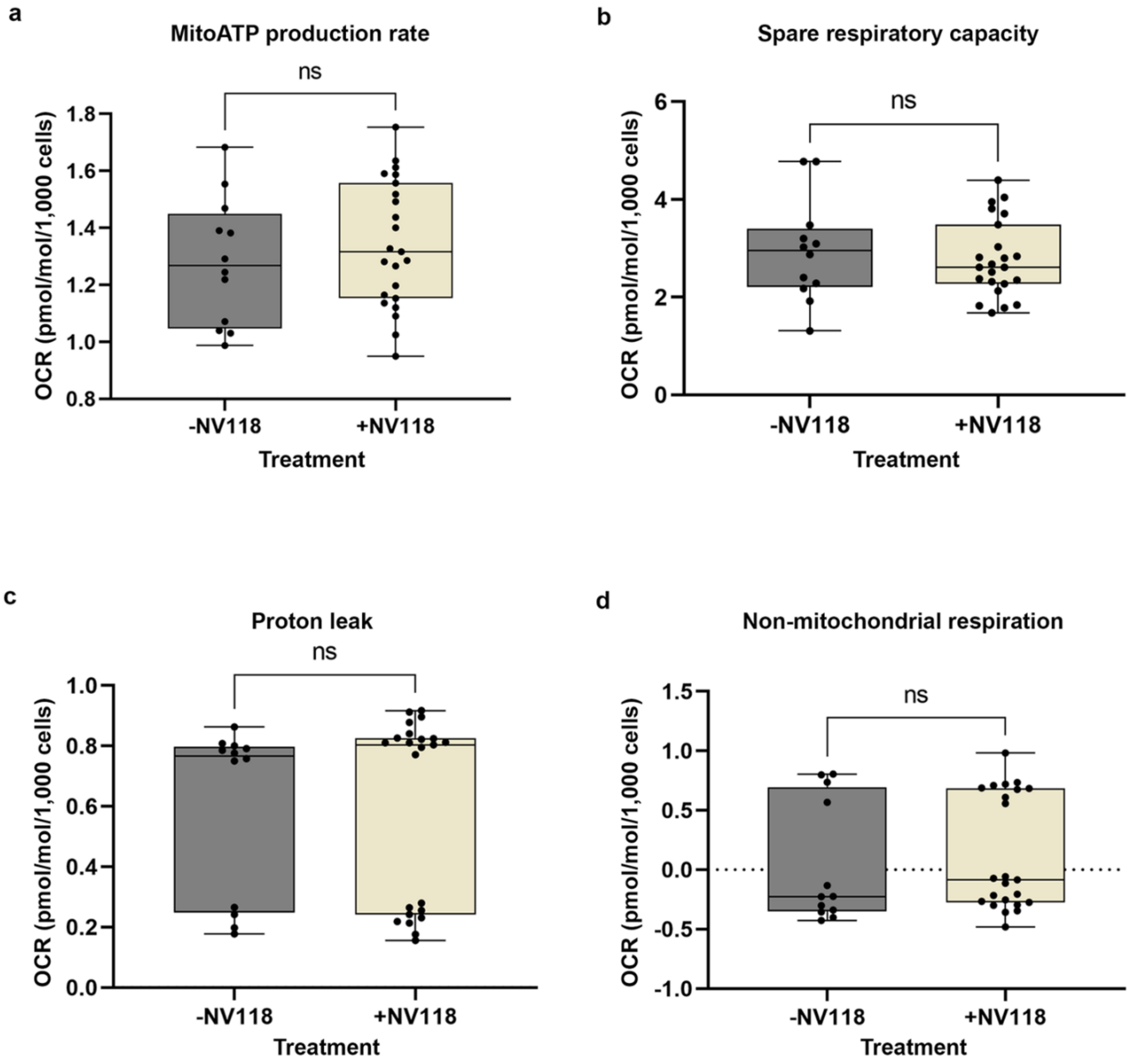


**Supplementary Figure S5. Mitochondrial respiration profile of CTL BJ-FB with and without NV118.** CTL BJ-FB were treated with 100 µM NV118 or vehicle (phenol-red free MEM) for 24-hours, and Oxygen Consumption Rate (OCR) was measured at the end of the 24-hour treatment period. Cell line showing (a) mitoATP production rate, (b) spare respiratory capacity, (c) proton leak, (d) non-mitochondrial respiration. All parameters are in pmol/min/1000 cells. Data are mean +/- SD. Experiments were repeated at least three times on different days under the same conditions. ns = not significant. The dark gray bar represents treatment with vehicle (-NV118), while the tan bar represents treatment with 100 µM NV118 (+NV118).

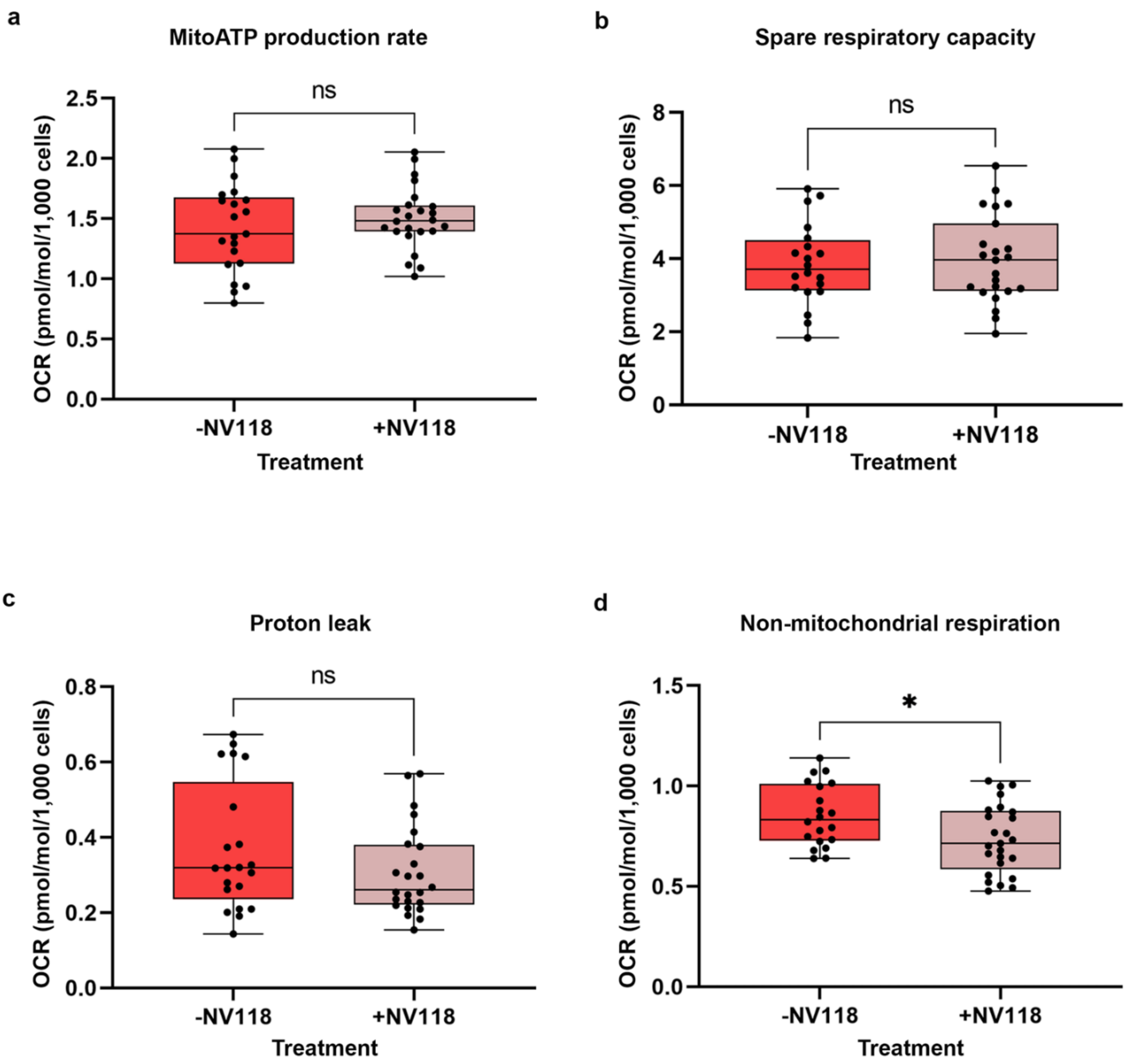


**Supplementary Figure S6. Mitochondrial respiration profile of SBG1-FB (*T8993G*) with and without NV118. SBG1-FB (*T8993G*)** were treated with 100 µM NV118 or vehicle (phenol-red free MEM) for 24-hours, and Oxygen Consumption Rate (OCR) was measured at the end of the 24-hour treatment period. Cell line showing (a) mitoATP production rate, (b) spare respiratory capacity, (c) proton leak, (d) non-mitochondrial respiration. All parameters are in pmol/min/1000 cells. Data are mean +/- SD. Experiments were repeated at least three times on different days under the same conditions. *p< 0.05, ns = not significant. The red bar represents treatment with vehicle (-NV118), while the pink bar represents treatment with 100 µM NV118 (+NV118).

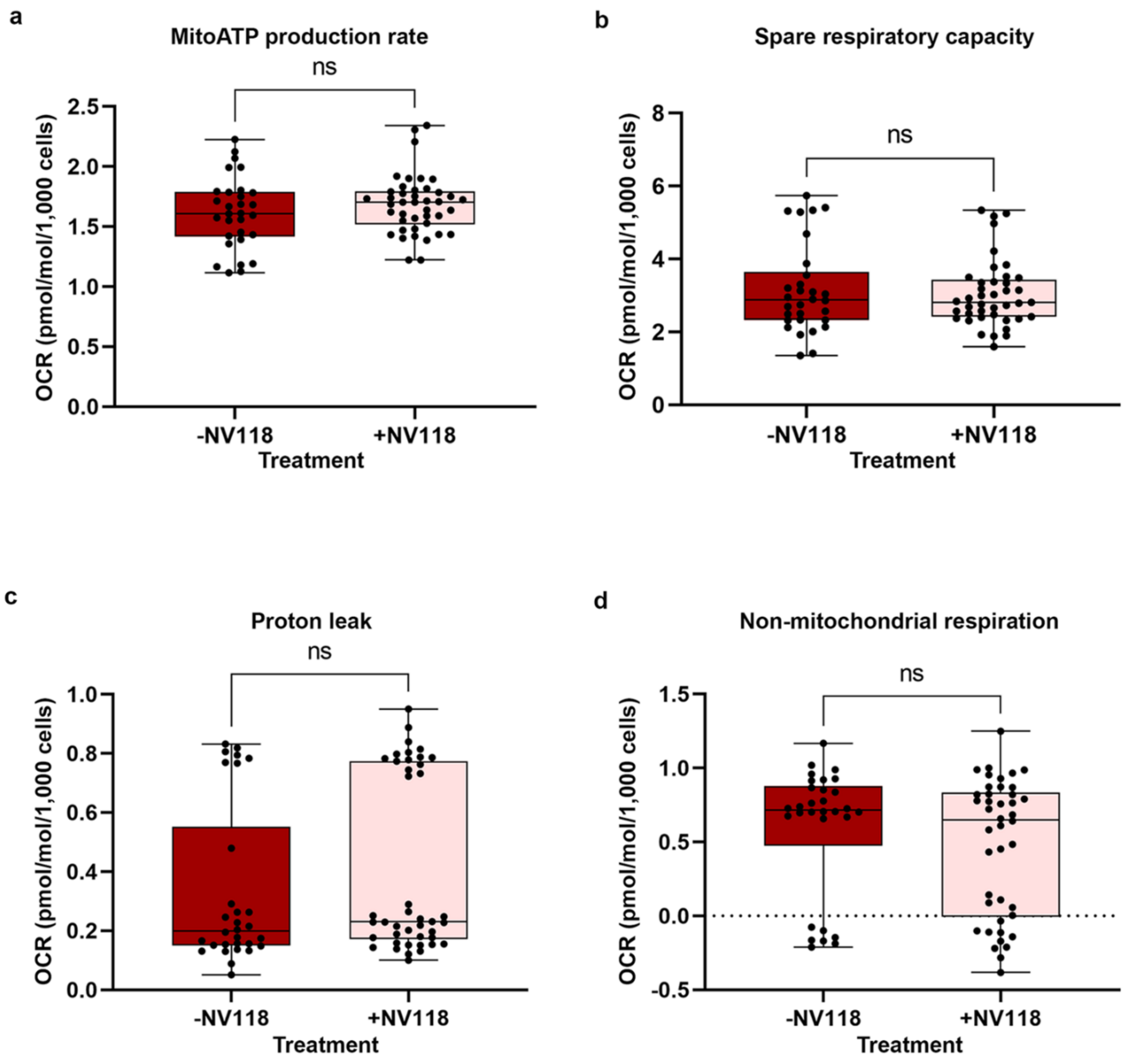


**Supplementary Figure S7. Mitochondrial respiration profile of SBG2-FB (*T8993G*) with and without NV118.** SBG2-FB (*T8993G*) were treated with 100 µM NV118 or vehicle (phenol-red free MEM) for 24-hours, and Oxygen Consumption Rate (OCR) was measured at the end of the 24-hour treatment period. Cell line showing (a) mitoATP production rate, (b) spare respiratory capacity, (c) proton leak, (d) non-mitochondrial respiration. All parameters are in pmol/min/1000 cells. Data are mean +/- SD. Experiments were repeated at least three times on different days under the same conditions. *$p < 0.05$, ns = not significant. Dark red bars represent treatment with vehicle (-NV118), while pink bars represent treatment with 100 µM NV118 (+NV118).

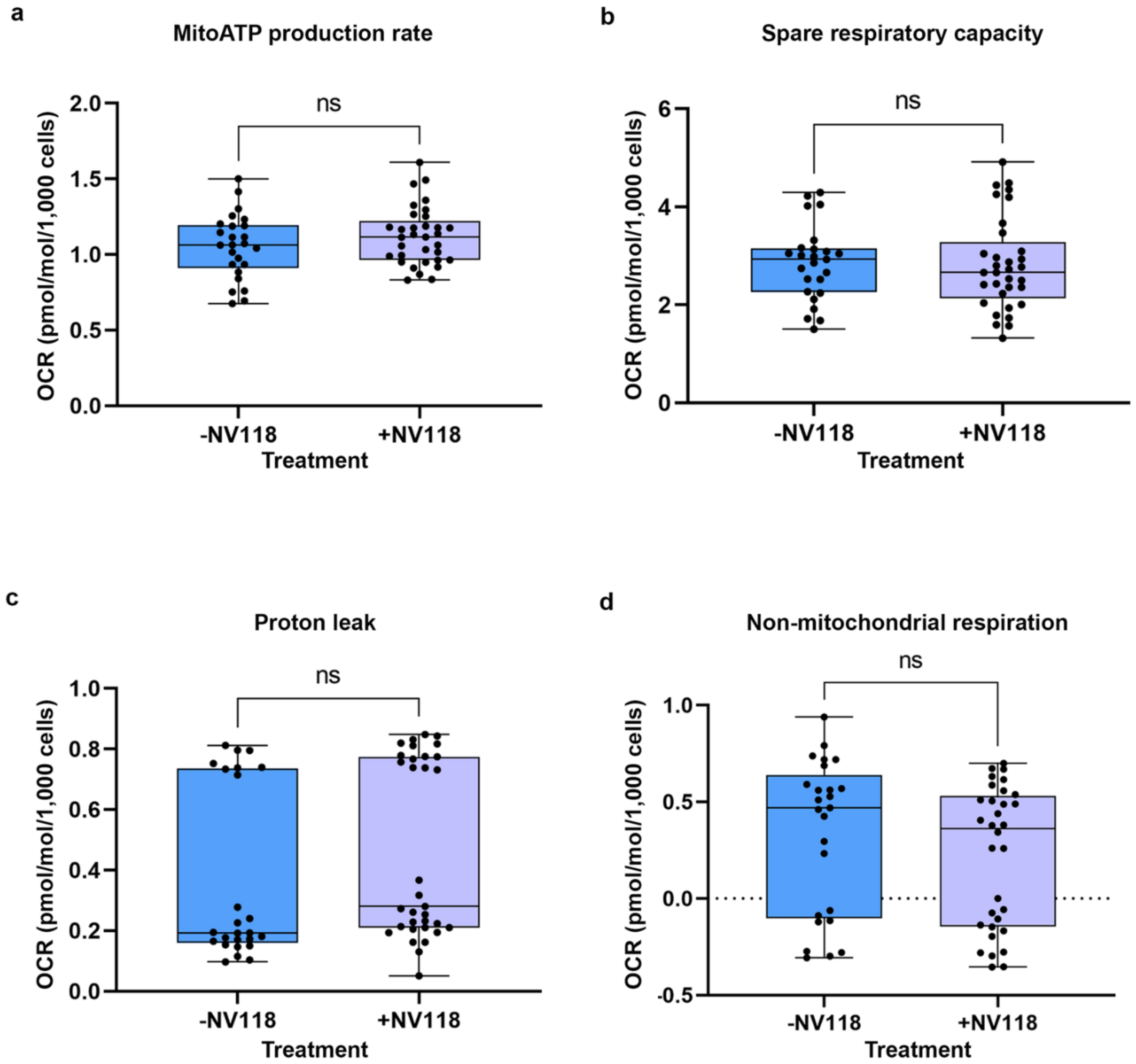


**Supplementary Figure S8. Mitochondrial respiration profile of SBG4-FB (*T10158C*) with and without NV118.** SBG4-FB (*T10158C*) were treated with 100 µM NV118 or vehicle (phenol-red free MEM) for 24-hours, and Oxygen Consumption Rate (OCR) was measured at the end of the 24-hour treatment period. Cell line showing (a) mitoATP production rate, (b) spare respiratory capacity, (c) proton leak, (d) non-mitochondrial respiration. All parameters are in pmol/min/1000 cells. Data are mean +/- SD. Experiments were repeated at least three times on different days under the same conditions. *p< 0.05, ns = not significant. Blue bars represent treatment with vehicle (-NV118), while purple bars represent treatment with 100 µM NV118 (+NV118).

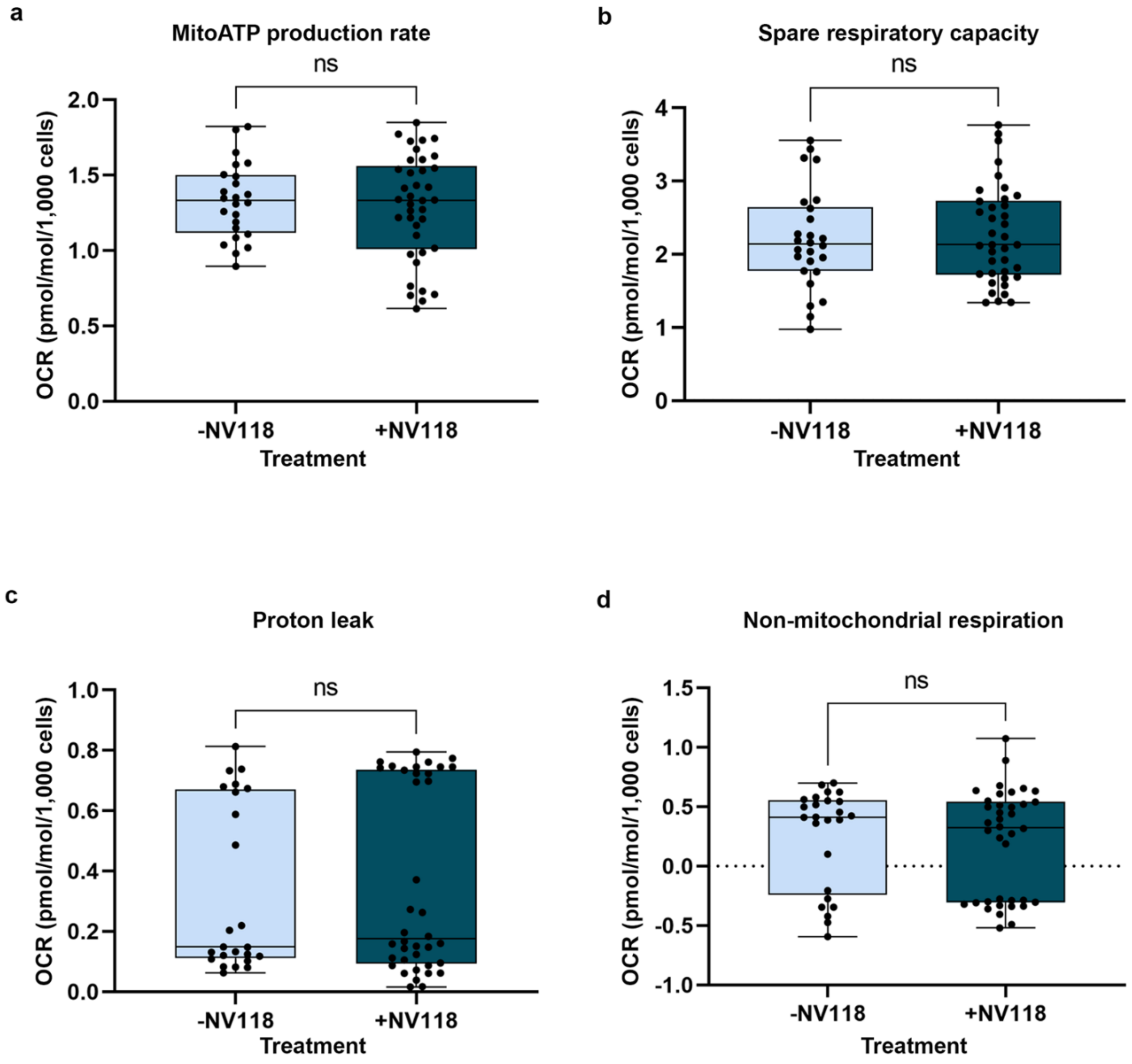


**Supplementary Figure S9. Mitochondrial respiration profile of SBG5-FB (*T12706C*) with and without NV118.** SBG5-FB (*T12706C*) were treated with 100 µM NV118 or vehicle (phenol-red free MEM) for 24-hours, and Oxygen Consumption Rate (OCR) was measured at the end of the 24-hour treatment period. Cell line showing (a) mitoATP production rate, (b) spare respiratory capacity, (c) proton leak, (d) non-mitochondrial respiration. All parameters are in pmol/min/1000 cells. Data are mean +/- SD. Experiments were repeated at least three times on different days under the same conditions. *p< 0.05, ns = not significant. Light blue bars represent treatment with vehicle (-NV118), while green bars represent treatment with 100 µM NV118 (+NV118).

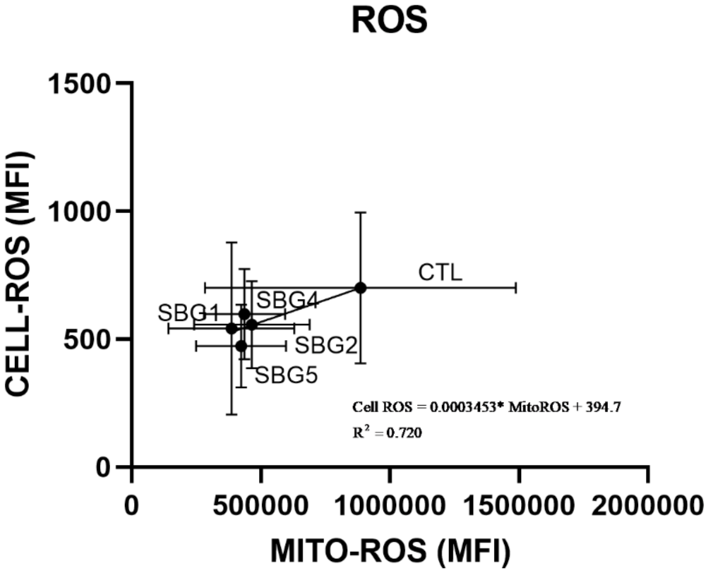


**Supplementary Figure S10.** Correlation plot between mitochondrial ROS and cellular ROS. As noted, there is a positive correlation between the two parameters, with increase in mitoROS correlating with an increase in cellular ROS.. Correlation data is plotted for all five lines (CTL, SBG1-FB, SBG2-FB, SBG4-FB, SBG5-FB).
